# Advancing Non-Atom-Centered Basis Methods for More Accurate Interaction Energies: Benchmarks and Large-Scale Applications

**DOI:** 10.1021/acs.jpca.4c04689

**Published:** 2024-11-18

**Authors:** Balázs D. Lőrincz, Péter R. Nagy

**Affiliations:** † Department of Physical Chemistry and Materials Science, Faculty of Chemical Technology and Biotechnology, Budapest University of Technology and Economics, Műegyetem rkp. 3., H-1111 Budapest, Hungary; ‡ HUN-REN−BME Quantum Chemistry Research Group, Müegyetem rkp. 3., H-1111 Budapest, Hungary; § MTA−BME Lendület Quantum Chemistry Research Group, Müegyetem rkp. 3., H-1111 Budapest, Hungary

## Abstract

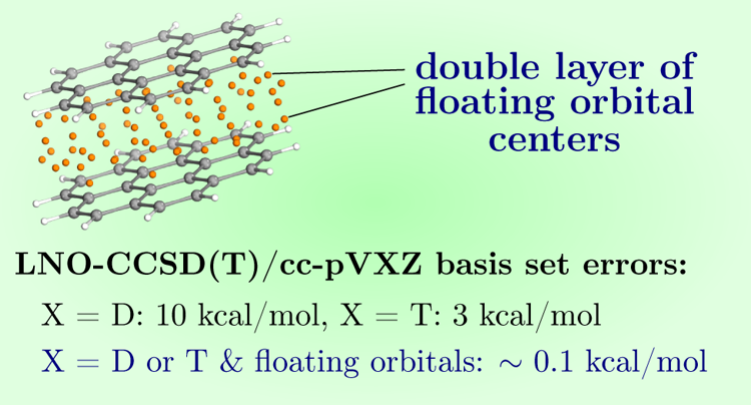

Recent advances in local electron correlation approaches have enabled the relatively routine access to CCSD(T) [that is, coupled cluster (CC) with single, double, and perturbative triple excitations] computations for molecules of a hundred or more atoms. Here, approaching their complete basis set (CBS) limit becomes more challenging due to extensive basis set superposition errors, often necessitating the use of large atomic orbital (AO) basis sets with diffuse functions. Here, we study a potential remedy in the form of non-atom-centered or floating orbitals (FOs). FOs are still rarely employed even for small molecules due to the practical complication of defining their position, number, exponents, etc. The most frequently used FO method thus simply places a single FO center with a large number of FOs toward the middle of noncovalent dimers; however, a single FO center for larger complexes can soon become insufficient. A recent alternative uses a grid of FO centers around the monomers with a single s function per center, which is currently applicable only for H, C, N, and O atoms. Here, we build on the above advantages and mitigate some drawbacks of previous FO approaches by using a layer of FO centers and 4–9 FOs/center for each monomer. Thus, a double layer of FOs is placed between the interacting subsystems. When extending the double-ζ AO basis with this double layer of FOs, the quality of conventional augmented double-ζ or conventional triple-ζ AO bases can be reached or surpassed with less orbitals, leading to few tenths of a kcal/mol basis set errors for medium-sized dimers. This good performance extends to larger molecules (shown here up to 72 atoms), as efficient local natural orbital (LNO) CCSD(T) computations with only double-ζ AO and 4 FOs/center FO bases match our LNO–CCSD(T)/CBS reference within ca. 0.1 kcal/mol. These developments introduce FO methods to the accurate modeling of large molecular complexes without limitations to atom types by further accelerating efficient correlation calculations, like LNO–CCSD(T).

## 1 Introduction

Noncovalent interactions play a major role across chemical sciences, such as in catalysis, surface, supramolecular, or biochemistry. For example, they can govern the mechanism, stereochemistry, or yield of chemical reactions, e.g., by affecting the structure and stability of transition state complexes. However, while the noncovalent interaction contributions are orders of magnitude smaller than covalent bond energies,^1−7^ their cumulative effect can be substantial, especially in extended systems.^8−13^ Hence, their accurate modeling is a challenging task necessitating advanced quantum chemistry tools. For example, the wave function-based electron correlation treatment could be reliable if combined with high-quality atomic orbital (AO) basis sets to approach their complete basis set (CBS) limit. Especially, the coupled cluster (CC) model^14−16^ with single and double excitations (CCSD)^17^ as well as contributions from triple excitations^18−20^ can provide systematically improvable results. Here, we employ the CCSD model with perturbative triple excitations [CCSD(T)],^20^ which is often referred to as gold standard in quantum chemistry.

While the CCSD(T) model was repeatedly shown to deliver chemical accuracy (i.e., <1 kcal/mol errors),^8,10,15^ its steep scaling limits its applicability range conventionally to ca. 20–25 atoms even with efficient parallel implementations.^21−28^ However, recent advances relying on, e.g., natural orbital (NO) based approximations^29−31^ and their combination with local correlation approaches^32−43^ extended the limits of reliable CCSD(T) computations to 40–50^44,45^ and even to 100s of atoms.^43,46−48^ In this work, we will employ our local natural orbital (LNO) method,^43,48−53^ which enabled so far some of the most advanced CCSD(T) computations for complex intermolecular interactions. Namely, with our LNO–CCSD(T) method, we reported tightly converged augmented quintuple-ζ computations for complicated supramolecular complexes of up to 132 atoms,^54^ surface binding on ionic crystals matching the quality and uncertainty of experiments,^55^ CBS limit interaction energies for ion-ligand complexes,^56^ and quadruple-ζ level protein–ligand interaction energies up to 1023 protein atoms.^43,48^

Hence, especially for such larger molecules, the slow convergence of the correlation energy toward the CBS limit also poses challenges. Considering interactions, one can note that the traditional AO basis sets are usually optimized for atoms and thus can be expected to be less effective for the intermolecular region. Hence, routinely, at least triple- or quadruple-ζ AO basis sets are required, often augmented with diffuse functions for well-converged interaction energies. This can often lead to oversaturation at the atomic positions and undersaturation in the interacting region. Although some basis families offer an extensive hierarchy of systematically improving sets, increasing the number of AOs in this way can rapidly lead to linear-dependency issues for large molecules. While explicit electron correlation methods can accelerate the basis set convergence also for CC computations,^57−63^ they are most helpful to model the electron–electron cusp and may still require extensive diffuse basis sets for accurate noncovalent interactions.^64^

In this study, we systematically benchmark and propose novel methods specifically designed to accelerate the basis set convergence of noncovalent interactions by using orbitals residing not only on the atomic positions. Depending on their specific purpose, such (mostly Gaussian orbital based) approaches were referred to in the literature as non-atom-centered, midbond, off-center, or floating orbital (FO) basis methods. For example, Tao and co-workers added a single midbond orbital center to the middle of the noncovalent bonds between helium and other noble gas-containing noncovalent dimers.^65−69^ Extending this midbond concept, Mester and Kállay added ellipsoidal Gaussian type orbitals to the center of covalent bonds.^70^ In this way, placing a midbond function halfway between two covalently bonded atoms can play the role of polarization functions, while placing midbond functions in the space between two noncovalently interacting monomers contribute to the description of intermolecular interactions. To highlight their property of not being centered on the atomic positions or the middle of bonds in all cases, we will give preference to the floating orbital denomination.

Despite their advantages, such FO basis functions are still rarely used in practice, as they have numerous additional parameters to be defined compared with the case of AO basis sets. Namely, their position, number, exponent, and angular momenta have to be determined, which in general requires a difficult, nonlinear optimization procedure. The complicated task of treating these as variational parameters was taken on so far only by Császár and Tasi for a few prototypical atomic and molecular systems of 1–3 atoms.^71^ On top of that, the non-atom-centered basis parameters also probably depend on the quality of the AO basis set and the type of the intermolecular interactions.

Because of these complications, the relatively simple, single midbond orbital approach is applied in almost all FO-based studies.^65−69,72−75^ Due to the use of only one FO center, a relatively large FO basis is employed that often contains at least 3 sets of s and p, 2 sets of d, and 1 set of f basis functions (briefly 3s3p2d1f or 3321). Using this setup, Tao and co-workers carried out MP4 calculations and concluded that a smaller AO and FO basis set (than the pure AO basis set) is sufficient to reach the same accuracy in the interaction energies. This simple FO center definition was also adapted by Szalewicz and co-workers to study different, biologically relevant noncovalent complexes (S22 test set).^73^ They calculated interaction energies in a composite MP2 and CCSD(T) scheme and added the FO basis to reduce the AO basis set needed for CCSD(T) calculations. Patkowski and co-workers combined this single FO center approach with explicitly correlated wave function methods.^74,76−78^

Recently, Høyvik and co-workers investigated various noncovalent interaction types, such as H-bonds, dispersion, and mixed interactions for smaller (up to 6 atoms in the A24 set) and medium-sized (up to 36 atoms in the S66 set) complexes.^75^ They pointed out the need for high angular momentum FOs when using a single midbond center. Compared to the above studies, Høyvik and co-workers also studied the effect of adding a second FO center for the medium-sized complexes, which resulted in a slight improvement of the interaction energy accuracy. Along the line of including more FO centers, Neogrády and co-workers employed an FO center grid surrounding the entire surface of the monomers.^79,80^ Their approach places a single s type basis function on each grid point, whose parameters are optimized so far only for H, C, N, and O atoms.

In this study, we first systematically compare the performance of the single midbond and the FO center grid type methods for various noncovalent interactions of medium-sized complexes up to 36 atoms, including H-bonds, ionic H-bonds, dispersion, and mixed interactions.^81,82^ We also present a novel FO approach, building on some of the advantageous and overcoming some of the unfavorable properties of previous FO approaches. Namely, we use more than one FO center strategically placed in a double layer formation between the monomers to cover the region of noncovalent interaction. Moreover, we found optimal compromises between the single s type and a large 3s3p2d1f base by using a 1s1p or 1s1p1d FO basis on each FO center of the double layer. In general, this double layer FO basis can improve cc-pVDZ interaction energies to the quality of aug-cc-pVDZ or cc-pVTZ with ca. 1.5–2 times less basis functions. Going toward larger systems of high practical relevance, the proposed double layer method is more generally applicable as it does not have atom-type limitation and overcomes the problem of diminishing FO contributions occurring with a single FO center. Moreover, the number of FOs increases only with the size of the interacting surface, which is much more favorable than the scaling of, e.g., adding diffuse AOs to all atoms. We also demonstrate the applicability of the double layer FO method in combination with our LNO–CCSD(T) method to reach larger systems. We show that the CBS limit LNO–CCSD(T)-level interaction energy of the parallelly displaced coronene dimer (72 atoms) can be approached to within ca. 0.1 kcal/mol by adding the proposed FO basis to double- or triple-ζ AO basis sets.

The paper is written as follows: in Section 2, we summarize each of the previously applied FO approaches in detail (Sections 2.1 and 2.2), as well as introduce our novel FO method (Sections 2.3–2.5). In Section 3, we introduce the technical details of the computations. In Section 4, we present an in-depth analysis for a few complicated systems (e.g., uracil dimer, Section 4.1), benchmark statistics for medium-sized dimers (Section 4.2), and a large-scale application (Section 4.3).

## 2 Methodology

The floating orbital basis sets have the following parameters to determine: number and spatial coordinates of FO centers, angular momenta, and exponents. These parameters of the FOs could also depend on the underlying AO basis. While the AO basis optimization is already a complicated, nonlinear process, it still has less degrees of freedom. The reason is that common Gaussian basis sets are mostly atom-centered and developed for each element independently (although one can note some exceptions aiming at smaller AO basis sets that were found promising for density functional theory (DFT) interactions^83−85^). In contrast, the number and center position of the FOs are also unknown parameters. Additionally, due to their role in modeling noncovalent interactions, the independent, element-wise optimization of the FO basis set parameters does not seem to be an ideal strategy. All in all, the global optimization of all FO parameters, including their position, AO basis, and molecular interaction dependence is a very challenging task, which probably contributed to the limited use of FO methods in computational chemistry. However, as shown in previous studies, it is not necessary to address all of the above complexities at once to define useful FO methods.^65,79^

**Figure 1:**
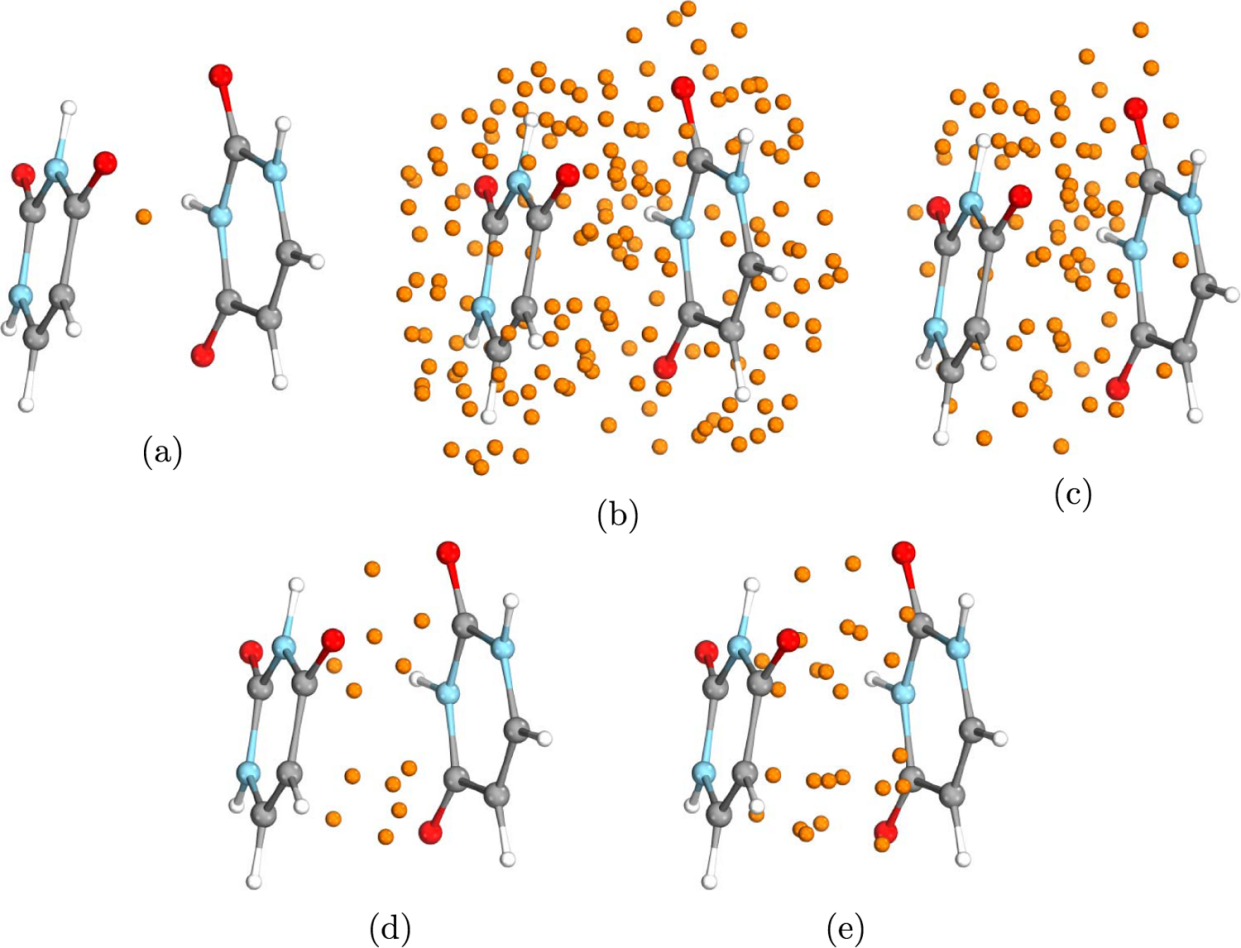
Position of floating orbitals (represented by the orange spheres) for the uracil dimer (24 atoms) of the S66 test set with various FO methods: weighted geometric center (a), monomer surface grid (b), interacting region grid (c), single layer (d), and double layer (e). The number of FO centers for these five methods is 1, 172, 78, 12, and 24, respectively.

First, we discuss and analyze the properties of the existing FO methods in more detail. To that end, in Table 1, a brief summary is given on the existing FO methods^65,79^ compared to our novel approach, with more details collected in Sections 2.1–2.5. In the most often employed approach of Tao and co-workers (detailed in Section 2.1), a single FO center is placed between the two interacting monomers (Figure 1a).^65^ In the early versions, the midpoint of the monomer center of masses was selected as the FO center. Later, Szalewicz and co-workers improved the definition of this single FO center position, which we review in Section 2.1 and will refer to here as weighted geometric center (WGC). The most commonly applied basis placed on that center consists of 3 sets of s, 3 sets of p, 2 sets of d, and 1–1 sets of f and g functions, which will be referred to as 3s3p2d1f1g or shortly 33211, collecting 38 basis functions altogether.^86^

**Table 1:** Summary of Floating Orbital Basis Method Parameters from Literature Studies as well as for the Method Introduced in This Work

floating orbital (FO) basis method	weighted geometric center (WGC)^65,87^	monomer surface grid (msG)^79^	double layer (DL)
FO basis	3s3p2d1f1g	1s	1s1p(1d)
number of FO centers	1	∼3–7× system size	∼interacting surface size
number of FOs/center	38	1	4 (9)
position of FOs	WGC of dimer	grid around monomers	interacting region
exponents	from ref (65)	optimized in ref (79)	adapted from ref (65)
applicability	dimers of small monomers	only H,C,N,O atoms	no atom type or size restriction

The WGC and related single FO center methods were employed so far for relatively small monomers,^65−69^ most recently going up to 18 atoms.^75^ This is partly explained by the previous computational limitations of CCSD(T), and thus, efforts were not yet devoted to testing or extending WGC-like approaches to larger molecules. However, we anticipate that FOs on a single FO center could only cover relatively small monomers (ca. 10–20 atoms) sufficiently well, while the efficacy of a single FO center could decrease with increasing monomer size. Moreover, the small monomers appearing in WGC-like applications also had a relatively simple structure and shape compared with the complexity emerging with increasing system size. While a small monomer having a flat or relatively spherical shape has a surface simple enough for satisfactory WGC definition, the proper placement of a single (or small number of) FO center(s) is more challenging for trimers, tetramers, etc., and for more complicated dimers, such as host–guest complexes. For example, if the host surrounds the guest molecule, the WGC definition could place the FO center somewhere within the space of the guest molecule, while the complicated shape of the surface where the host and guest interact could prevent the identification of key position(s) for the placement of a single (few) FO center(s).

An alternative, second approach for the FO center definition was introduced by Neogrády and co-workers,^79^ overcoming the use of only a single FO center. They placed a grid of FO centers on the surfaces of the interacting monomers. While they did not introduce a name for their approach, in this comparative study, we will refer to it as the monomer surface grid (msG) method (detailed in Section 2.2). This FO center definition based on monomer surfaces is expected to be more general than WGC, as it is applicable to complexes with a wide range of sizes, shapes, and monomer number. As they employed a relatively dense grid (with somewhat smaller grid edge length than covalent bond lengths), the number of FO centers in the msG method is approximately 3–7 times larger than the number of atoms in the dimer (Figure 1b). The FO basis of the msG method was defined using a single s type function (1s) per msG FO center. Using this setup, they optimized the msG model parameters determining the FO positions and exponents for representative molecular dimers containing H, C, N, and O atoms.^79^ Therefore, currently, the msG parameters are defined only for these four elements and in the corresponding limited chemical space.

In this study, we introduce a third approach, which will be referred to as the double layer (DL) method (Figure 1e). The goal of the DL method is to extend the applicability range of previous FO methods and improve their performance. To that end, we identify the beneficial features of the WGC and msG methods, generalize, and combine them with novel ideas (Table 1). In brief (details in Section 2.5), we place a layer of FO centers on the surface of each noncovalently interacting monomer only on the surface side facing the other monomer. The number of DL FO centers significantly extends the single center of WGC, but it is considerably smaller than the number of msG FO centers (cf. Figure 1b,e) because we focus on the intermonomer region that plays an important role in the noncovalent interaction. The DL FO centers are placed so that almost all of the atoms on the surface have a dedicated FO center. Regarding the FO basis placed on each FO center, we appreciate the larger than 1s basis sets used in the WGC method and the larger number and more strategically positioned FO centers of the msG approach. In the DL method, we combine these directions by using less FO centers (than in msG) with more FOs per center, including 1s1p and 1s1p1d contributions with exponents taken from the WGC method.^65^ The resulting basis definition of the DL method does not require optimization and thus can be employed for more general complexes without restrictions to the elements constituting the monomers.

To better explain these aspects, next, the three investigated methods (and some variants of them) are introduced in detail in Sections 2.1–2.5.

### 2.1 Weighted Geometric Center (WGC) Method

The FO method of Tao and co-workers, which is used most often in practice defines the center of a single FO halfway between the center of mass of each subsystem.^65−69^ This approach was first proposed for the study of noble gas-containing dimers and then was extended to monomers of up to 3–4 atoms. Turning to more complicated cases with monomers of markedly different size, such as the helium-cyanoacetylene dimer, Szalewicz and co-workers found that the FO center definition of Tao et al. could lead to a midpoint placed closer to one of the monomers.^87^ When this occurs, the FOs on the single FO center are more beneficial to the description of that closer (larger) monomer. To overcome this asymmetry, Szalewicz and co-workers proposed an improved FO center placement, where the position [**r**_WGC_ in eq 1] is the *r*^–6^ weighted average of the midpoints of intermonomer atom pairs
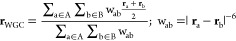
1Here, the *w*_ab_ weight is the inverse sixth power of the atom–atom distances of monomers A and B, while **r**_a_ and **r**_b_ are the spatial coordinates of atoms from subsystems A and B, respectively. To reference this definition, we will call this method here as the weighted geometric center (WGC) approach.

The motivation behind the *r*^–6^ weights in eq 1 is the analogous decay of dispersion interactions between two atoms on different monomers. Consequently, larger weights are assigned to more strongly interacting atom pairs that are closer to each other. Therefore, the definition of eq 1 does not favor the larger monomer and incorporates information about the monomer surfaces and their atoms. For example, in Figure 1a, the WGC of the uracil dimer is located in the position with the smallest intermolecular distances, slightly shifted from the midpoint between the centers of the six-membered rings.

Originally, Tao and co-workers employed 3s, 3p, 2d, and 1f (shortly 3s3p2d1f or 3321) basis functions on a single FO center. The exponents of these FOs were determined in ref (65). This 3321 FO basis was later extended with a g function by Christiansen et al. when investigating the benzene-argon dimer (resulting in 3s3p2d1f1g or shortly 33211 FO basis).^86^ Due to the excellent performance of the 33211 FO basis, recently it was adapted by Høyvik and co-workers.^75^ They found that for smaller systems (below 6-atom monomers), the results obtained via the single FO center are more convincing than for larger systems (below 18-atom monomers) in combination with double-ζ AO basis sets. Thus, Høyvik and co-workers also manually added a second FO center for the larger monomers, based on chemical intuition, which led to moderate improvements over the results with a single FO center. These limited set of results for medium-sized dimers suggests that the use of a single (or two) FO center(s) could not be sufficient when studying even larger molecular complexes.

### 2.2 Monomer Surface Grid (msG) of Floating Orbital Centers

The approach of Neogrády and co-workers defines a grid of FO centers placed onto the surface of each monomer (monomer surface grid, msG method).^79,80^ The FO centers are determined in three steps for each subsystem summarized briefly as follows

1. The subsystem surface is defined as the union of spheres around each atom of the subsystem with a radius of r_msG_.
2. A uniform grid of points (with grid edge length e_msG_) is projected onto the subsystem surface separately from the three directions defined by the principal axes of the subsystem.
3. Revision/removal of grid points that are too close to each other to avoid linear dependency in the combined AO and FO basis set:
  (a) removal of one of the intramonomer grid points from those that are too close to each other
  (b) offset of intermonomer grid point pairs that are close to each other

For example, the msG FO center list generated this way for the uracil dimer is presented in Figure 1b.

The parameters of the msG approach (*r*_msG_, *e*_msG_, and the exponent of the s type FO) were optimized in ref (79) for the H, C, N, and O atom types. The msG parameters were optimized for the interaction energies of representative systems from the S22 test set: stacked and hydrogen-bonded uracil dimer and stacked benzene-indole complex with both equilibrium and partly dissociated geometries. To simplify the optimization procedure, only two parameter sets were determined, one set for hydrogen and another set for non-hydrogen (C, N, and O) atoms. However, as the FO parameters are available only for these four atom types, the current applicability of the msG method is limited to the corresponding chemical space. While the FO center positions are not as simple to obtain as for the WGC approach, both the WGC and msG FO center coordinates translate and rotate with the molecular orientation, providing independence from the choice of the coordinate system.

As the optimum of the *e*_msG_ grid edge length turned out to be smaller than 1 Å for the four atom types, usually multiple FO centers are assigned to each atom on the monomer surface. Therefore, the number of centers are 3–7 times larger than the number of atoms in the dimer. The combination of this msG FO basis with AO bases not including diffuse AOs provided similar performance for the studied interactions as the same AO basis without FOs, but augmented with diffuse functions both in terms of numerical performance and basis set size (that is, e.g., cc-pVDZ + msG vs aug-cc-pVDZ).^79,80^

### 2.3 Interacting Region: Space between the Monomers

Considering the msG FO centers, e.g., in Figure 1b, not every FO center is expected to be equally important for the interaction energy: those FO centers that are placed between the interacting monomers could be more important than the others outside of the space between the monomers. To investigate this assumption further, let us identify those atoms of the surface of each subsystem that could be more important for the interaction. This interacting surface atom list will be used in Section 2.4 to place FO centers located only between the monomers.

**Figure 2:**
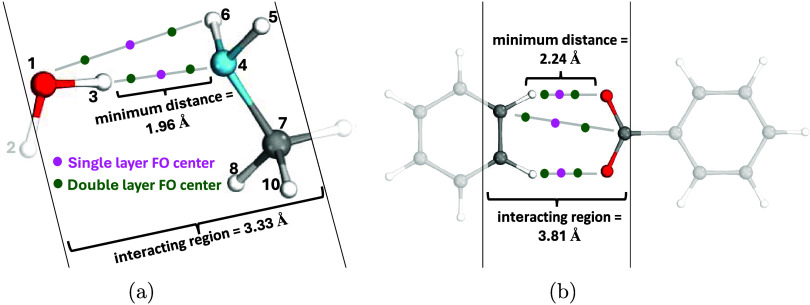
Illustration of the interacting surface atom list definition as well as the positions of floating orbital centers for the single layer (pink dots) and the double layer (green dots) methods for the water–methyl-amine H-bonded complex (a) and for the benzene–benzoate ionic H-bonded complex (b).

As the majority of the noncovalent interaction components is expected to originate from the atoms and electrons residing on the surface of the interacting monomers, we focus on the space between the monomers, simply referred to as the interacting region here. We determine the atom list of the interacting surface as follows (Figure 2):

1. Measure the minimum distance (MD) between two monomers
2. MD is multiplied by a scaling factor (SF > 1). This scaled minimum distance controls the spatial extent of the interacting region.
3. Intermonomer atom pairs with a distance lower than SF·MD are selected to constitute the interacting surface.

For example, for a smaller (S) and a larger (L) fragment, let *s*_*i*_ and *l*_*j*_ label the *i*th and *j*th atom of S and L, respectively. Then, with *d*_*ij*_ being the distance between the *s*_*i*_–*l*_*j*_ atom pair, this atom pair is added to the interacting surface if *d*_*ij*_ < SF·MD.

This approach is illustrated in Figure 2 on the examples of the water–methyl-amine hydrogen-bonded complex (Figure 2a) as well as on the benzene–benzoate ionic hydrogen-bonded complex (Figure 2b). For the sake of brevity, we give only a detailed description of the interacting surface definition for the smaller, water–methyl-amine dimer. Here, MD = 1.96 Å, which is the distance between the hydrogen atom (number 3) of water and the nitrogen atom (number 4) of methyl-amine. Then, the spatial extent of the interacting region, SF·MD = 1.70·1.96 Å, becomes 3.33 Å, where SF = 1.70 was determined based on the inspection of the retained interacting surface atom list for S66 with multiple SF choices. To the first water atom (oxygen, number 1), atoms 4, 5, and 6 of methyl-amine are closer than SF·MD; thus, first, atoms 1, 4, 5, and 6 are added to the interacting surface. Then, for atom 3 of water, atoms 7, 8, and 10 of methyl-amine are added, while none of the methyl-amine atoms are closer to atom 2 of water than SF · MD.

### 2.4 Interacting Region Grid (irG) of Floating Orbital Centers

Utilizing the interacting surface atom list of Section 2.3, we propose to restrict the FO center list of msG to an interacting region between the subsystems. To that end, we identify the msG centers that are sufficiently close to the atoms of the interacting surface in the subsystems as follows:

1. We select the subsystem with the smaller number of atoms on the interacting surface. For the *i*th atom on the interacting surface subset of this subsystem, we find its closest intersubsystem atom pair, with distance *d*_*ij*_. Then, we use these average of the *d*_*ij*_ distances to define the average distance between the interacting surface parts on the two subsystems (*D*).
2. If a msG FO center is closer to any interacting surface atom than *D*, then we add this FO center to the interacting region grid (shortly irG).

For example, for the uracil dimer, *D* = 3.24 Å, the number of FO centers in the irG is 78 (Figure 1c), which is less than the number of msG FO centers in Figure 1b by 94 centers, or 55%. While the irG method considerably reduces the number of FO centers to about 3-times the number of dimer atoms, the irG centers are still closely packed. This FO center density might be necessary if only a single s type FO is placed on each irG center. Thus, in Section 2.5, we also investigate if less FO centers are sufficient in combination with somewhat more FOs/FO center.

### 2.5 Single and Double Layer of Floating Orbital Centers

To find a more compact FO center list, let us recognize that in the irG approach, there are practically two densely packed FO grids placed near the interacting region part on the two subsystems. Building on this, we propose two options, directly placing a single and a double layer (SL and DL) of FO centers into the interacting region. The centers of the SL (DL) method are determined so that we assign roughly one (one pair of) FO center(s) to one intersubsystem atom pair as follows:

1. For the subsystem with the smaller number of atoms on the interacting surface, we go through its surface atoms and find the closest intersubsystem atom pair for each. The pairing is made to be a bijection, starting with the smallest intersubsystem distance (minimum distance, MD in Figure 2).
2. The SL (DL) FO centers are placed to the midpoints (trisection points) of the intersubsystem atom pairs of step 1 (see Figure 2).

SL (DL) definition is illustrated in detail in Figure 2 on the water–methyl-amine H-bonded dimer. The first atom pair (atoms 3 and 4) corresponds to the MD. Due to the bijective construction, each atom can be utilized only once; thus, atoms 1 and 6 define the second atom pair. As the smaller interacting surface (corresponding to the water molecule) consists of only 2 atoms, only two (four) SL (DL) FO centers are constructed in this example.

The choice of not using a surface atom more than once for the SL/DL definition is useful to avoid close lying FO centers and to construct a relatively even distribution of FO centers. For the SL (DL) methods, there is roughly one FO center for each surface atom pair (surface atom), which is considerably less than that for the irG approach. Hence, we can consider placing more than a single s type function on the SL/DL FO centers, while the total number of FOs remains similar to irG or even less than with the msG method. A potential benefit of SL over DL is the somewhat fewer additional FOs, although compared to the size of the AO basis set, both the SL and DL FO numbers are relatively small. On the other hand, we prefer DL over SL, especially when the dimer distance is longer (e.g., for larger monomers in Section 4.3 or for monomers of more irregular shape) or the monomers are, e.g., somewhat dissociated, as SL would place the FOs to a larger distance from the monomers in such cases.

In our numerical analysis, we assess FO bases of increasing size: 1s, 1s1p, 1s1p1d, and 2s2p1d. We introduce the shorthand notation of the method/FO basis, e.g., DL/1s1p, for these combinations. For the sake of comparability with the WGC method, the exponents of these FOs were taken from ref (65). Where there are more exponents to the same angular momentum in the 3s3p2d1f1g basis of ref (86), the most diffuse exponents were retained. For example, when constructing the 1s1p1d FO basis building on 3s3p2d, the 0.1 exponent (in atomic units) was retained from the s exponent list of 0.9, 0.3, and 0.1.

## 3 Computational Details

Density fitting (DF)-based conventional CCSD(T),^28^ local MP2,^49,50^ and local natural orbital (LNO)-based^43,48,51−53^ local CCSD(T) computations have been performed using the 2023 version of the Mrcc quantum chemistry program suite.^88,89^ Pople type 6-31+G(2d)^83^ and Dunning type correlation consistent basis sets^90,91^ with and without diffuse functions [(aug-)cc-pVXZ, X = D, T], as well as heavy augmented basis sets (haug-cc-pVXZ, X = D, T), were employed which are abbreviated as [(h)aug]XZ in the figures of the manuscript and the Supporting Information (SI).

DF approximation was utilized for every computation with the corresponding DF auxiliary bases for the HF^92^ and correlation energy^93^ calculations, that is, (aug-)cc-pVXZ-RI-JK and (aug-)cc-pVXZ-RI were employed with the (aug-)cc-pVXZ AO basis set. Szalewicz and co-workers adapted the 3s3p2d1f FO basis from the work of Tao et al. and proposed the 5s5p5d4f3g DF auxiliary basis.^94^ Later, Christiansen and co-workers introduced the 3s3p2f1f1g FO basis,^86^ but they did not define a DF auxiliary basis corresponding to their 3s3p2d1f1g FO basis. Therefore, we extended the 5s5p5d4f3g basis with 3 sets of h functions in this work, which resulted in a 5s5p5d4f3g3h auxiliary basis. Moreover, DF auxiliary bases 2s1p-RI, 3s2p1d-RI, and 4s3p2d1f-RI were constructed to fit the 1s, 1s1p, and 1s1p1d (2s2p1d) FO bases, respectively. The same FO DF auxiliary basis was employed for both the HF and the correlation energy calculations. The exponents of these FO auxiliary bases are also available in the SI.

For the complete basis set (CBS) extrapolation of the HF energies, the two-point formula suggested by Karton and Martin is used with the recommended parameters.^95,96^ Conventional and LNO-based correlation energies were extrapolated with the formula of Helgaker and co-workers^97^ with an exponent of 2.46 (2.51) for the (aug-)cc-pV(D,T)Z extrapolation and an exponent of 3 for the (aug-)cc-pV(T,Q)Z extrapolation.

The structures of the S66 compilation were taken from the original work of Řezáč et al.,^81^ while the 21 selected ionic H-bonded dimer structures were taken from the IHB100 test set compiled by Řezáč.^82^ The calculations were performed on equilibrium dimer structures for both the S66 and the IHB100 test set. The names of the selected 21 ionic H-bonded dimers are given in the SI. We utilized MP2-F12/aug-cc-pV(T,Q)Z-F12 and MP2/aug-cc-pV(Q,5)Z interaction energies as CBS references for the S66 and IHB100 DF-MP2 interaction energies, respectively.^82,98^

To characterize the performance of the different FO methods, we calculated the mean absolute error (MAE), the root-mean-square deviation (RMSD), and the maximum absolute error (MAX). The timing measurements are performed with a single 64-core AMD EPYC 7763 processor.

## 4 Results and Discussion

The accuracy assessment and comparison of the previous and here introduced FO methods depend on a large variety of aspects. Besides the noted FO parameters (number and position of FO centers, and basis set parameters, like exponents), such a benchmark work should consider the AO basis combined with the FO method types, the level of electron correlation treatment, as well as the representative molecule and noncovalent interaction types. Here, we consider the following:

1. FO methods: weighted geometric center (WGC), monomer surface grid (msG), interacting region grid (irG), single layer (SL), and double layer (DL)
2. FO basis sets: 1s, 1s1p, 1s1p1d, 2s2p1d, 3s3p2d1f1g
3. AO basis sets: 6-31+G(2d), (h)aug-cc-pVXZ, and cc-pVXZ, X = D, T
4. wave function methods: MP2, CCSD(T)
5. noncovalent interaction types: hydrogen bond, dispersion, mixed, ionic hydrogen bond

The number of variables to be considered is too large to explore and discuss here all possible combinations. Therefore, first we show in detail three of the most complicated dimers of the S66 test set,^81^ that is the uracil dimer (π–π stacking), the uracil base pair, as well as the benzene-peptide dimer to illustrate the more important trends, and to decrease the number of setting combinations to be assessed (Section 4.1). Then, a comprehensive statistical analysis is presented for the more practical setting combinations on the S66 test set as well as on 21 ionic hydrogen-bonded complexes selected from the IHB100 test set^82^ (Section 4.2). Finally, a large-scale practical application is presented in Section 4.3.

### 4.1 Numerical Performance of FO Methods: Uracil Dimer

**Figure 3:**
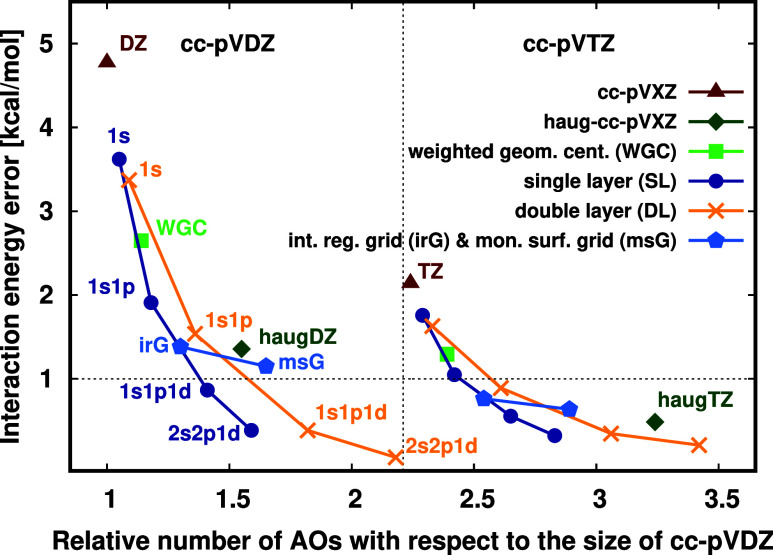
DF-CCSD(T)/cc-pVXZ (+FO) (X = D, T) correlation energy contribution error of CP corrected interaction energies as a function of the combined AO and FO basis set size for the π–π stacked uracil dimer of the S66. Reference: CP corrected DF-CCSD(T)/haug-cc-pV(T,Q)Z.^98^

We start the analysis with the dispersion dominated (π–π stacking) uracil dimer from the S66 test set, as this system exhibits the largest, more than 5 kcal/mol basis set incompleteness error (BSIE) in the interaction energy with the cc-pVDZ basis set. Moreover, we carried out similar studies on the uracil base pair and the benzene-peptide dimer, the results of which are available in the SI (Figures S1 and S2). Additionally, we found that the basis set errors are analogous for MP2 and CCSD(T). Here, the CCSD(T) results are presented (e.g., in Figure 3 for the π–π stacking uracil dimer), while the analogous MP2 results are available in the SI (Figure S2). We also combined the FO methods with the 6-31+G(2d) AO basis set,^83−85^ which results are available in Table S1 in the SI.

The improvement of the BSIE for various FO methods with respect to the CBS limit reference [that is, CP corrected CCSD(T)/aug’-cc-pV(T,Q)Z] is demonstrated in Figure 3, in combination with the cc-pVDZ (left panel) and the cc-pVTZ AO basis sets (right panel). Here, we show on the *x* axis the total number of AO and FO basis functions relative to the size of cc-pVDZ. Apparently, the rate of improvement due to the increasing number of additional FO functions is similar for cc-pVDZ, cc-pVTZ, and 6-31+G(2d). Therefore, we will mainly focus on the results with the cc-pVDZ AO basis.

Starting with the most conventional WGC/3s3p2d1f1g FO method, the error of interaction energy decreases by 40% for the dispersion dominated uracil dimer (green square in Figure 3) and decreases by 50 and 60% for the uracil base pair and benzene-peptide dimer, respectively (green squares in Figure S1a,b) compared to the pure cc-pVDZ results. The monomer surface grid method already achieves haug-cc-pVDZ accuracy for all three investigated dimers (c.f. msG vs haugDZ in Figures 3 and S1) in line with the results of ref (79). Moreover, the cc-pVDZ + msG results outperform those with the cc-pVTZ AO basis set, using 1.3–1.4 times less basis functions for all three cases, but the improvement gained via the msG method is somewhat more pronounced for the π–π stacked uracil dimer and for the benzene-peptide dimer than for the uracil base pair. Compared to the msG results, retaining only the FOs in the interacting region, the irG approach leads to negligible loss of accuracy with about half of the basis functions, for all three systems.

Turning to the analysis of the single layer (SL) and the double layer (DL) FO methods (dark blue and orange curves in Figures 3, S1 and S2, respectively), the effect of systematically adding higher angular momentum functions in the interacting region can also be studied. The convergence trend toward the CBS limit is analogous for all three dimers, when comparing the SL and the DL FO methods; therefore, we will mainly discuss the DL results. Starting with the π–π stacked uracil dimer, its interaction energy error with the pure AO basis decreases by only 20% when DL/1s is employed. By adding DL/1s1p to cc-pVDZ, the accuracy of cc-pVTZ, while with cc-pVDZ + DL/1s1p1d even the accuracy of haug-cc-pVTZ is reached with only 61 and 56% of the basis functions, respectively. The interaction energy error decreases even further with the DL/2s2p1d method; however, the majority of the improvement occurs already with 1s1p and 1s1p1d. This implies that the further extension of the FO basis might not lead to notable improvements, at least in combination with double- and triple-ζ AO basis sets. Finally, comparing the FO methods to each other, DL/1s1p and irG considerably outperform WGC by providing haug-cc-pVDZ quality with fewer orbitals than in haug-cc-pVDZ. Compared to these, the msG basis moderately, while DL/1s1p1d basis significantly improves the results, the latter outperforms even cc-pVTZ + WGC, and matches haug-cc-pVTZ and cc-pVTZ + msG with significantly smaller number of basis functions altogether.

Inspecting the results of the uracil base pair, the improvement gained via the SL and DL methods is less significant with respect to the pure cc-pVDZ AO basis set than for the π–π stacked uracil dimer. For example, to reach the accuracy of the pure cc-pVTZ or haug-cc-pVDZ AO basis set, at least the cc-pVDZ + DL/1s1p1d FO basis is necessary, while none of the here introduced FO bases added to cc-pVDZ seems to approach the quality of the pure haug-cc-pVTZ. The dependence of the SL and DL results on the interaction types can be attributed to the different sizes of the interacting surface atom lists. Namely, the near parallel placement of the monomers in the π–π stacking uracil dimer results in half of the dimer atoms (12 atoms in this case) in the interacting surface atom list. Compared to that, for the uracil base pair, only the two H-bond donor and two acceptor atoms are added to the interacting surface atom list, which brings less FOs to the interacting region altogether, thus improving BSIEs to a smaller extent.

### 4.2 Statistical Analysis on Noncovalent Dimer Test Sets

We continue with a statistical analysis on the representative S66 set, separated into its conventional H-bonding, dispersion, and mixed (polar, dispersion, etc.) interaction subsets. We found it important to extend the benchmark set with stronger interactions and shorter intermolecular distances to make sure that the FO centers are not too close to each other in such cases. To that end, we selected 21 representative ionic H-bonded complexes from the IHB100 test set, referred to as IHB100/21.^82^ Due to the similarity of the basis set convergence of the MP2 and CCSD(T) interaction energies for the uracil dimer (c.f. Figures 3 and S2), we expect that an MP2-based statistical analysis on S66 and IHB100/21 will also be representative for the case of CCSD(T). Therefore, to enable the assessment of a large number of AO and FO combinations, we continue with an MP2-based error analysis.

**Figure 4:**
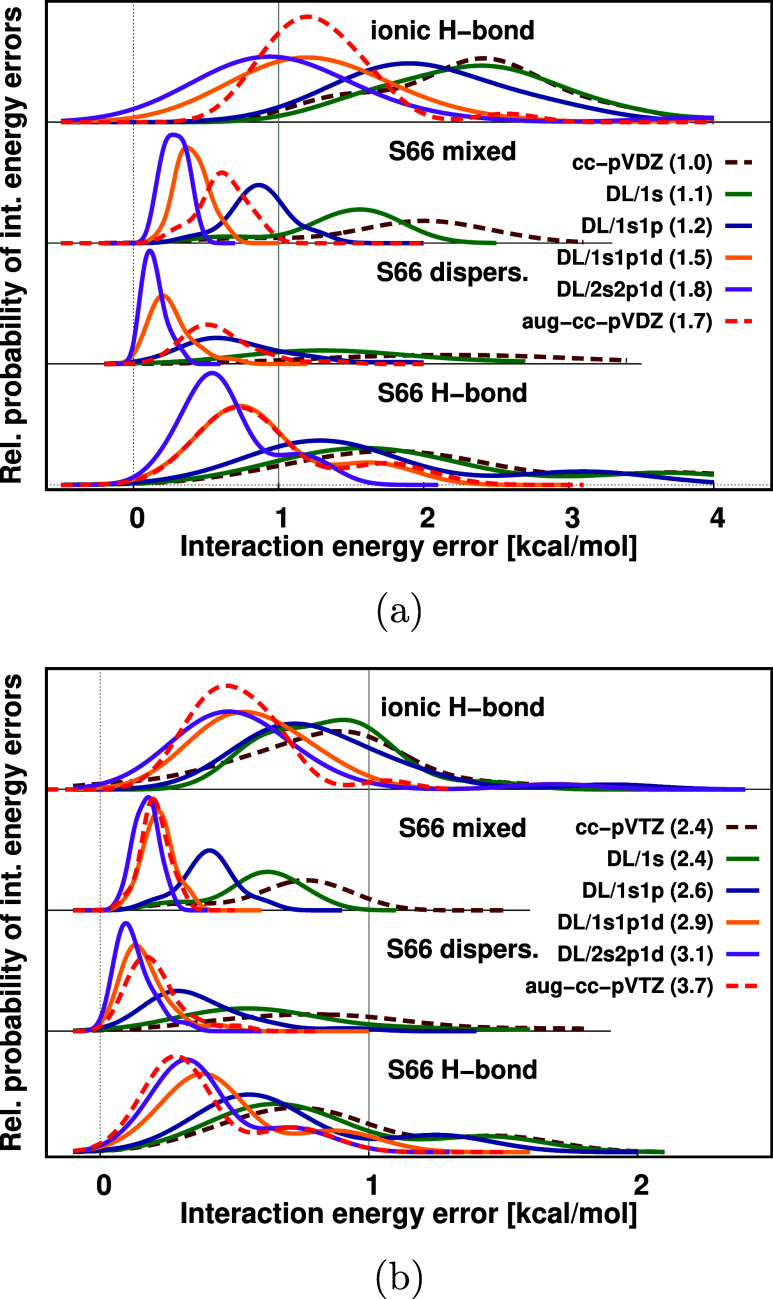
Relative probability of interaction energy errors with the double layer FO method and 1s, 1s1p, 1s1p1d, and 2s2p1d FO bases separated to four subsets. (The analogous single layer FO results are plotted in Figure S3 of the SI). Level of theory: DF-MP2/(aug-)cc-pVXZ, with X = D in panel (a) and X = T in panel (b). The total number of basis functions relative to the size of cc-pVDZ is collected in parentheses besides the basis set labels.

First, we investigate the performance of the DL method in Figure 4 with respect to increasing the FO basis size. Here, we present the distribution of interaction energy errors for the DL FO method added to the cc-pVDZ (Figure 4a), cc-pVTZ (Figure 4b), and aug-cc-pVDZ (Figure S3c of the SI) conventional AO basis sets, respectively. (The analogous single layer results are collected in Figure S3a,b of the SI). Similarly to the case of the uracil dimer (Section 4.1), the convergence toward the CBS limit when adding more FOs is analogous with the cc-pVDZ, cc-pVTZ, and also with the aug-cc-pVDZ AO basis sets for each interaction type; therefore, we will discuss in detail only the case of cc-pVDZ (Figure 4a).

For the hydrogen-bonded dimers of S66 (bottom panels of Figure 4), two peaks are found on the error distribution curves, around 2 and 4 kcal/mol. The second peak at 4 kcal/mol can be attributed to overcorrections caused by the Counterpoise correction for double H-bonded systems of S66: uracil base pair, acetic acid dimer, acetamide dimer, acetic acid-uracil dimer, and acetamide-uracil dimer. This overcorrection has been pointed out for H-bonded systems of S66 previously.^98^ The size of the BSIE with cc-pVDZ is decreased by the additional FOs, diffuse functions, and the increased AO basis set (c.f. aug-cc-pVDZ in Figure 4a, and cc-pVTZ or aug-cc-pVTZ in Figure 4b); however, this second peak remains also with the largest basis set employed here (aug-cc-pVTZ).

Extending the pure cc-pV*X*Z AO basis (dark red-dashed curves in Figure 4) with diffuse functions (light red-dashed curves in Figure 4) as well as with FO basis of increasing size (solid curves in Figure 4) affects noncovalent bond types differently: the H-bonded dimers in S66 and IHB100/21 form one, and the dispersion dominated and mixed systems of S66 form a second set. Comparing the AO basis and the DL/1s performance (dark red-dashed and green curves of Figure 4), we again find a marginal improvement that is somewhat more pronounced for the dispersion/mixed subsets. Adding the DL/1s1p FOs (blue curves) to cc-pVDZ (cc-pVTZ) already brings the peaks of the error distributions to around 1 (around 0.5) kcal/mol, with somewhat larger errors remaining for the H-bonded cc-pVDZ + DL/1s1p cases. These numerical results can also be studied in Tables 2 and in S2 of the SI, where mean absolute error (MAE) values are collected for the four molecular subsets as well as for the various AO and FO settings separately. A second step of significant improvement comes from adding the d functions of DL/1s1p1d (orange curves). Here, one finds 0.91 (1.41) and 0.48 (0.63) kcal/mol MAEs with cc-pVDZ + DL/1s1p1d and cc-pVTZ + DL/1s1p1d settings, respectively, for H-bonds (ionic H-bonds) in the top of Table 2 (bottom of Table S2 of the SI). Similarly significant improvement is observed for dispersion (mixed) interactions, where 0.26 (0.41) as well as 0.17 (0.31) kcal/mol MAEs characterize the numerical performances of the cc-pVDZ + DL/1s1p1d and cc-pVTZ + DL/1s1p1d, respectively, as seen in the bottom of Table 2 (top of Table S2 of the SI). The cc-pVDZ + FO results outperform the pure cc-pVTZ errors for most dimers already with DL/1s1p, and for the ionic H-bonds with DL/1s1p1d. Moreover, the cc-pVDZ + DL/1s1p1d basis delivers comparable performance to aug-cc-pVDZ for the H-bonded subsets and outperforms the aug-cc-pVDZ AO basis for the dispersion/mixed subsets with a somewhat smaller basis set in average. Compared to that, again, the improvement brought by DL/2s2p1d (purple curves in Figure 4) is systematic, but relatively small.

**Table 2:**
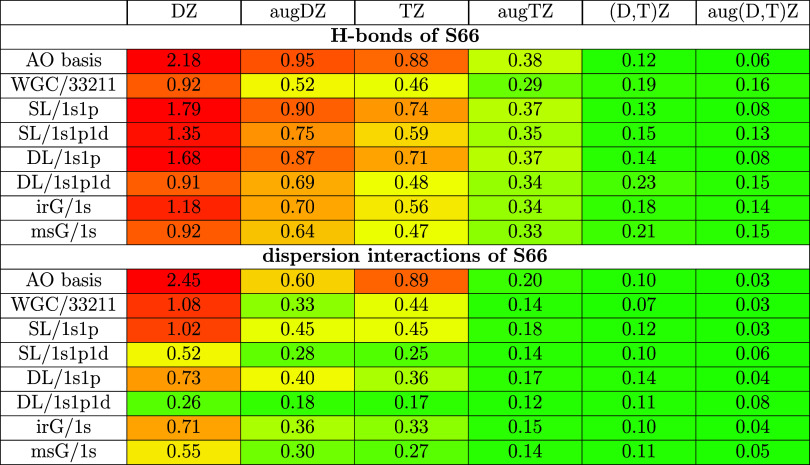
Mean Absolute Basis Set Errors (kcal/mol) for the H-Bonded (Top) and Dispersion Dominated (Bottom) Subsets of S66^a^

a Level of theory: DF-MP2/(aug-)cc-pVXZ; X = D, T.

Inspecting the number of FOs added to the dimers of H-bonded subsets and to the other two S66 subsets provides an additional layer of understanding to their somewhat different behavior. Namely, the interacting surface for the H-bonded dimers often consists of the H-bond donor and acceptor atoms (often resulting in 2 atoms for single and 4 atoms for double H-bond interacting surfaces). Thus, the corresponding FO basis for 1s and 1s1p contain not much more than 2–8 FOs. Compared to that, the 5 FOs per center extension brought by the d set of 1s1p1d is significant. In contrast, the dispersion/mixed interacting surfaces are larger, which yield more FO centers. Moreover, the dispersion interactions are significantly weaker, which often means smaller absolute BSIE. The contribution of the above factors provides additional explanation to why we observe faster convergence toward the CBS limit reference with increasing FO size for the dispersive/mixed subsets than for the H-bonded dimers.

**Figure 5:**
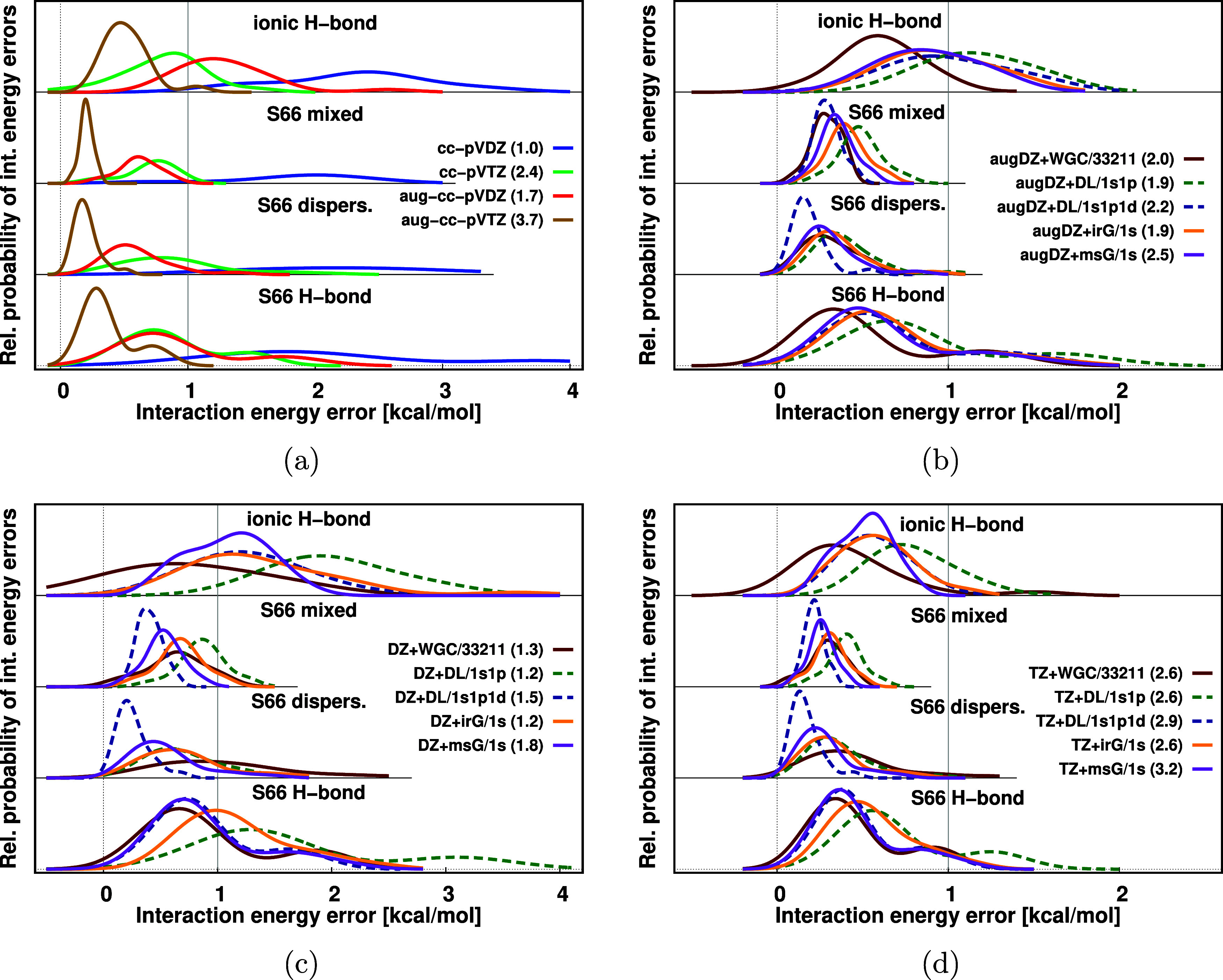
Relative probability of interaction energy errors at DF-MP2/(aug-)cc-pVXZ, X = D, T level of theory using pure AO basis sets (a) and various FO bases added to cc-pVDZ (DZ, c), aug-cc-pVDZ (augDZ, b), and cc-pVTZ (TZ, d). The total number of basis functions relative to the size of cc-pVDZ is collected in parentheses besides the basis set labels.

As the DL method (especially with the 1s1p and 1s1p1d basis) is the more accurate protocol compared to the SL in Figure S4 of the SI, the numerical performance of DL/1s1p and DL/1s1p1d will be investigated in Figure 5 more thoroughly, we set aside the 1s, 2s2p1d, and larger FO basis set options. In Figure 5, we compare the performance of different AO basis sets (cc-pVXZ and aug-cc-pVXZ; X = D, T in Figure 5a) with that of the FO methods extending the cc-pVDZ (Figure 5c), aug-cc-pVDZ (Figure 5b), and cc-pVTZ (Figure 5d) AO basis sets for the same four noncovalent interaction subsets. Let us briefly consider first only the pure AO basis sets. For the H-bonded systems (top and bottom panels of Figure 5a), the interaction energies improve more if the cardinal number is increased (from X = D to X = T, cf. blue and green curves), while for the dispersion/mixed subsets (two middle panels), the interaction energies improve more by adding diffuse functions (cf. blue and red curves).

Turning to the analysis of the WGC method (dark red curve) for H-bonded subsets, it has numerical performance comparable to those of irG, msG, and DL/1s1p1d methods (orange, purple, and blue dashed curves, respectively) for both the cc-pVDZ (Figure 5c) and the cc-pVTZ cases (Figure 5d). Moreover, combining these FO methods with the cc-pVDZ AO basis set, the accuracy of cc-pVTZ can be reached. Compared to that, the aug-cc-pVDZ + WGC/33211 combination (Figure 5b) slightly outperforms the other methods, probably due to the small size (up to 24 atoms) of the studied H-bonded dimers. In contrast to the case of the H-bonded subsets, we found a notably smaller improvement of the interaction energy errors with cc-pVDZ + WGC/33221 for dispersion/mixed systems as those subsets contain larger monomers and larger interacting surfaces. For comparison, the switching from cc-pVDZ AO basis set to aug-cc-pVDZ and cc-pVTZ (without FOs) for these dispersion (mixed) interactions already reduces the MAEs from 2.45 (1.83) kcal/mol to 0.60 (0.61) and 0.89 (0.70) kcal/mol, respectively (see Tables 2 and S2). Thus, the MAE of 1.08 (0.64) kcal/mol for cc-pVDZ + WGC/33211 is outperformed by pure aug-cc-pVDZ and cc-pVTZ, but adding the WGC to aug-cc-pVDZ or cc-pVTZ still almost halves their MAEs (Tables 2 and S2).

Considering the performance of the irG and msG methods in Figure 5c,5d (orange and purple curves), the maxima of their error distribution curves are already within ca. 0.2 kcal/mol of each other with a cc-pVDZ AO basis. This is achieved with 1.5 times less basis functions in the irG approach than the msG for each molecular subset. This difference in their local maxima is decreasing further with larger AO basis sets. These results again suggest that the most significant improvement can be achieved via adding FO centers to the interacting region first. We can also observe that the irG and msG performances are closer to each other for dispersion/mixed interactions, than for H-bonds, which also can be explained by the larger spatial extent of the interacting region. Since the interacting surface atom lists are more extended for dispersion/mixed systems, more FO centers are retained for these than for H-bonded dimers. Therefore, the BSIE values of the dispersive and mixed subsets with the irG method can become smaller.

Investigating the double layer method for dispersion/mixed interactions, the results with cc-pVDZ + DL/1s1p (dashed green curves in Figure 5) are comparable to those with the irG and msG methods, since a sufficient number of FO centers are added to the interacting region. This trend remains for the case of aug-cc-pVDZ and cc-pVTZ as well. Regarding the H-bonded systems with the smaller number of atoms on the interacting surfaces, DL/1s1p FOs bring smaller improvement, which trend is the most notable for cc-pVDZ and decreases with larger AO basis sets (aug-cc-pVDZ or cc-pVTZ). The next step of improvement comes from adding d-type FOs. Namely, cc-pVDZ + DL/1s1p1d (dashed blue curves in Figure 5) approaches the accuracy of aug-cc-pVTZ with 2.5 times less basis functions on average for dispersion/mixed interactions. Moreover, it is as good as or often notably outperforms msG and irG for all studied AO basis sets. Furthermore, the DL/1s1p1d approach provides comparable results to irG and msG methods for the H-bonding subsets.

**Figure 6:**
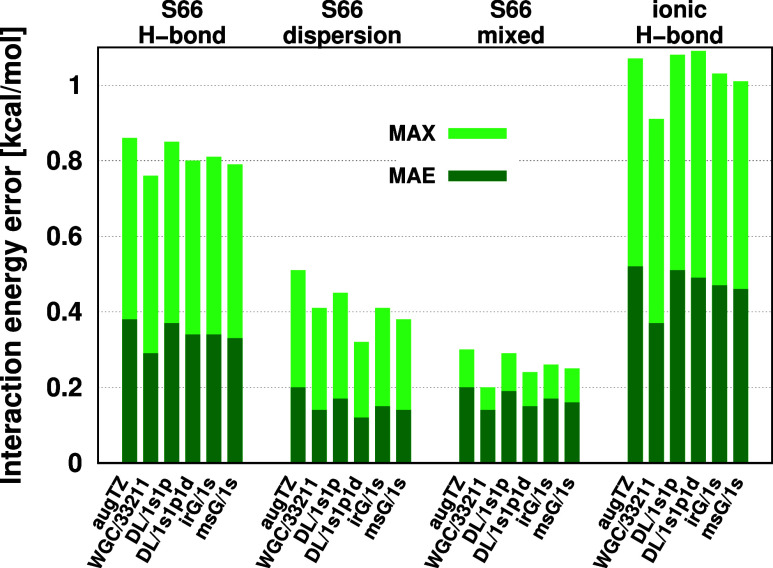
DF-MP2/aug-cc-pVTZ correlation energy contribution error of CP corrected interaction energies for the studied FO methods.

As we can observe in Figure 5c,5d (and also in Figure S5a–f of the SI), the error distribution curves for different FO methods overlap with each other more often as we extend the AO basis set size. The corresponding MAE values show the same, monotonic decrease (c.f. DZ, augDZ, TZ, and augTZ columns of Table 2). Since the smaller aug-cc-pVTZ errors are not visible well at this scale, we present their MAE and MAX values in bar charts (Figure 6). As these error measures are very close to each other with and without the additional FOs, here the efficiency of FO methods with larger AO basis sets (e.g., aug-cc-pVTZ) , appears to decrease. Explicitly, the improvements for all four investigated noncovalent interaction types and all FO methods is below ca. 0.1 kcal/mol with respect to the pure aug-cc-pVTZ results.

Here, the diffuse AOs in aug-cc-pVTZ probably span a space similar to the relatively low angular momentum orbitals in the FO basis, and thus the already small errors with respect to the CBS reference do not decrease considerably. Since the errors are already at the few tenths of a kcal/mol range, studying the effect of higher angular momentum FOs is set aside for future work.

We look more closely at the MAEs of the H-bonded and dispersion dimers of S66 in Table 2, while the analogous mixed and ionic H-bonded subset results are available in Table S2 of the SI. The MAEs also show that adding FOs to AO basis sets without diffuse AOs provides diffuse basis set quality results, especially with the msG, irG, and DL/1s1p1d FO methods. For example, cc-pVDZ + FO methods match pure aug-cc-pVDZ and cc-pVTZ results (cf. columns DZ–TZ of Table 2). Similarly, cc-pVTZ + FO results are comparable to the quality of aug-cc-pVTZ. These observations hold for WGC/33211 only for the H-bonded and for DL/1s1p only for the dispersion/mixed cases.

**Figure 7:**
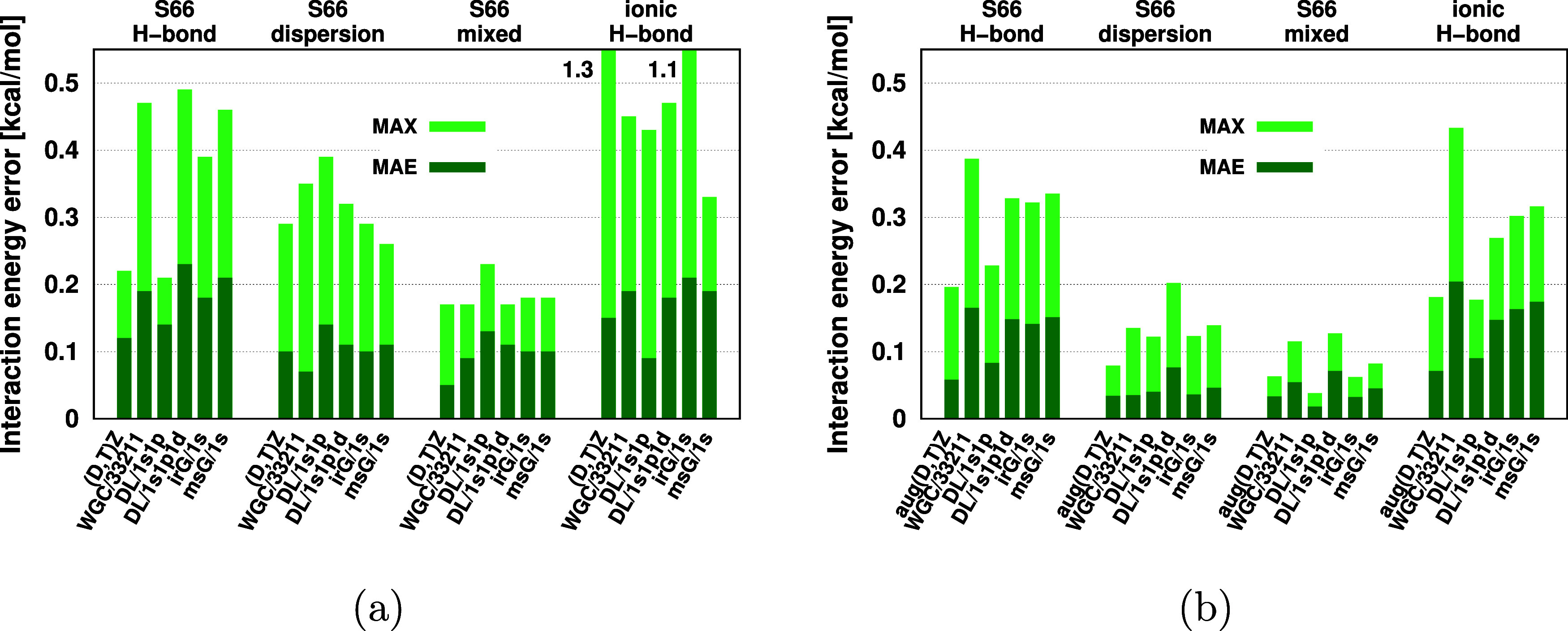
cc-pV(D,T)Z [(D,T)Z, a] and aug-cc-pV(D,T)Z [aug(D,T)Z, b] correlation energy contribution error of CP corrected interaction energies utilizing extrapolation exponent γ = 2.46 (2.51) for the (aug-)cc-pV(D,T)Z extrapolation.

We also carried out CBS extrapolations for AO basis sets with and without diffuse functions (last two columns of Table 2 and Figure 7). Partly because dispersion/mixed interactions are weaker than H-bonds in the studied cases, the corresponding cc-pV(D,T)Z [aug-cc-pV(D,T)Z] interaction energies are better converged for dispersion/mixed subsets than for H-bonds. Namely, in Figure 7, the MAEs for dispersion/mixed interaction energies are within 0.15 [0.10] kcal/mol, while for H-bonds, they are within 0.25 [0.20] kcal/mol, respectively. The extrapolated interaction energies improved only moderately or not at all using the presented FO methods with respect to the pure AO basis extrapolations (in terms of either MAX or MAE values).

**Figure 8:**
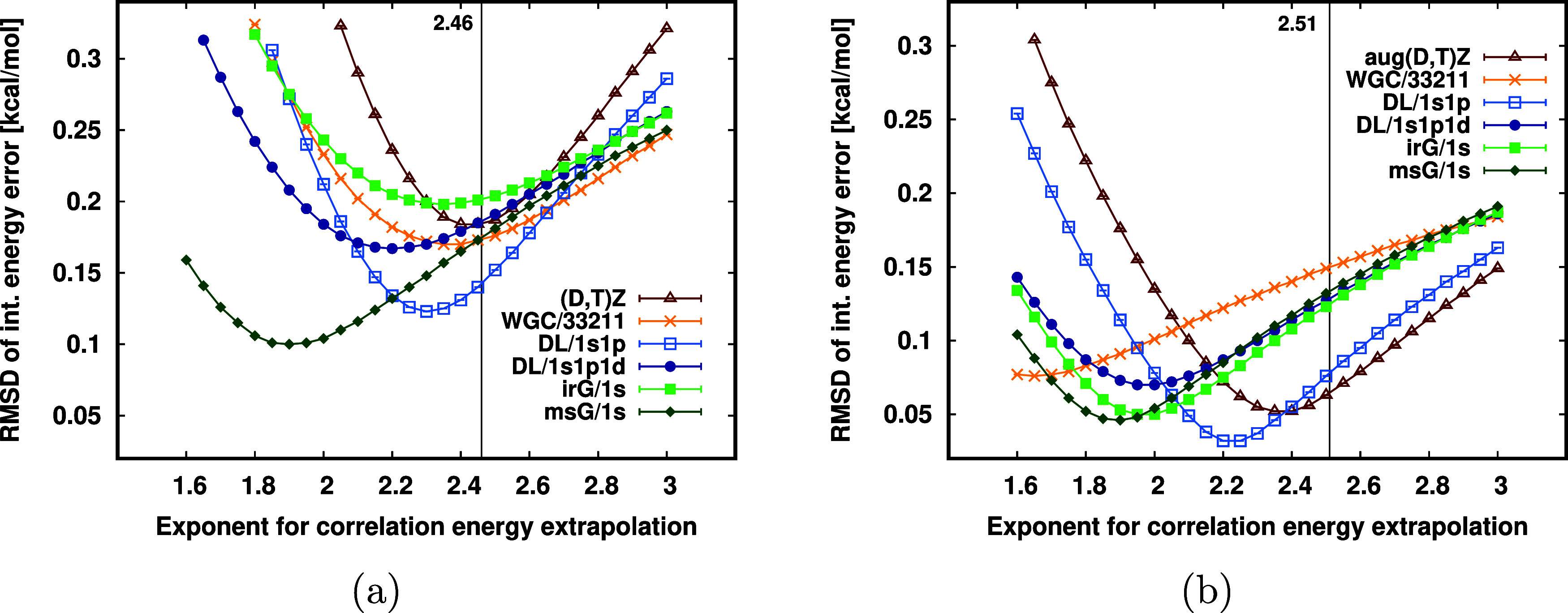
CBS extrapolation exponent scans for the cc-pV(D,T)Z [(D,T)Z, (a)] and aug-cc-pV(D,T)Z [aug(D,T)Z, (b)] extrapolation with various FO methods. Vertical lines at 2.46 and 2.51 on the *x* axis represent the previously recommended cc-pV(D,T)Z and aug-cc-pV(D,T)Z extrapolation exponents.^97^

Regarding the CBS extrapolations at the cc-pV(D,T)Z level, let us recall that the conventional inverse cubic extrapolation formula turned out to be less effective for smaller AO basis sets due to the slow correlation energy convergence toward the CBS limit at this basis set size.^97^ Therefore, empirical optimization of the extrapolation exponent γ = 3 was recommended, which resulted in γ = 2.46 for cc-pV(D,T)Z and γ = 2.51 for aug-cc-pV(D,T)Z.^97^ In Figure 8, we investigate if these empirical exponents still remain valid with the FO methods by scanning the interaction energy errors as a function of the extrapolation exponents in the range of [1.6,3.0]. Inspecting these exponent scans for the cc-pV(D,T)Z (Figure 8a) and aug-cc-pV(D,T)Z (Figure 8b) cases, the minima of the interaction energy error curves change only slightly. Explicitly, the results fall into a roughly 0.1 kcal/mol-wide range with exponents from 1.8 to 2.8 for both the cc-pV(D,T)Z and the aug-cc-pV(D,T)Z. This suggests that retaining the previously recommended exponents results in negligible loss of accuracy and we do not need to reoptimize CBS extrapolation expressions for each new FO method.

### 4.3 Large-Scale Application of the FO Methods

**Figure 9:**
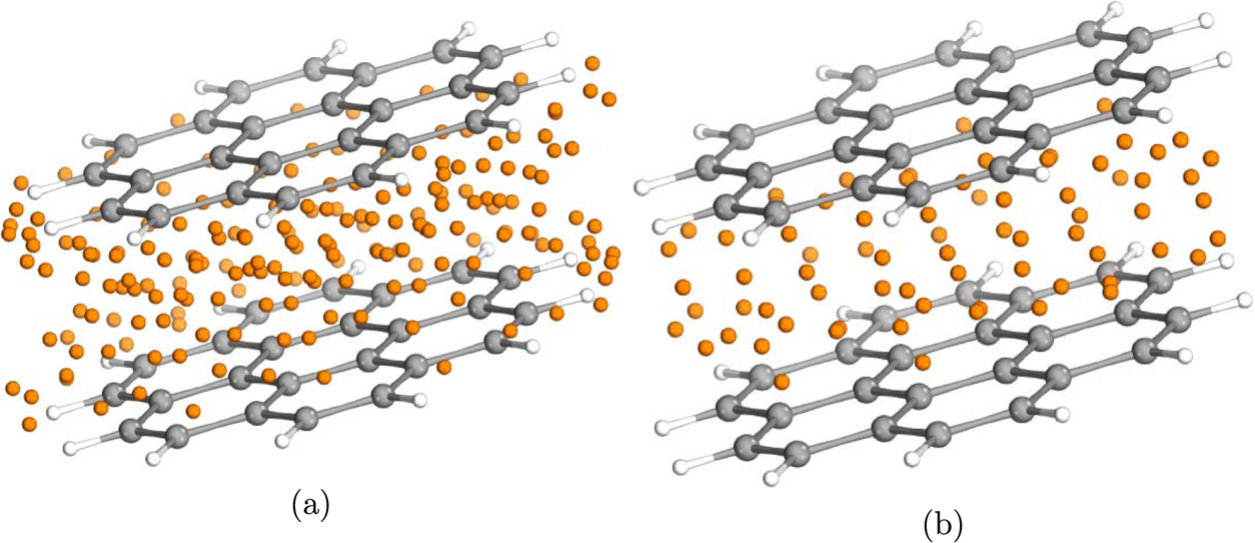
Position of the interacting region grid (irG, a) and the double layer (DL, b) FO centers for the parallelly displaced coronene dimer (represented by the orange spheres). Number of FO centers is 191 and 72, respectively.

In the previous sections, we focused on medium-sized molecular dimers (up to 36 atoms) to assess the previous and here proposed FO methods. However, one goal of the FO methods introduced here is the applicability to large molecules of practical interest. Therefore, we investigated the interaction energy convergence of the parallelly displaced coronene dimer (72 atoms, Figure 9), as this system requires diffuse, at least triple- or quadruple-ζ quality atom-centered basis sets and Counterpoise corrections for well-converged interaction energies according to our comparisons to aug-cc-pV(Q,5)Z level computations.^54^ However, while feasible with our LNO–CCSD(T) implementation, aug-cc-pVQZ computations are already quite demanding for large molecules.

**Figure 10:**
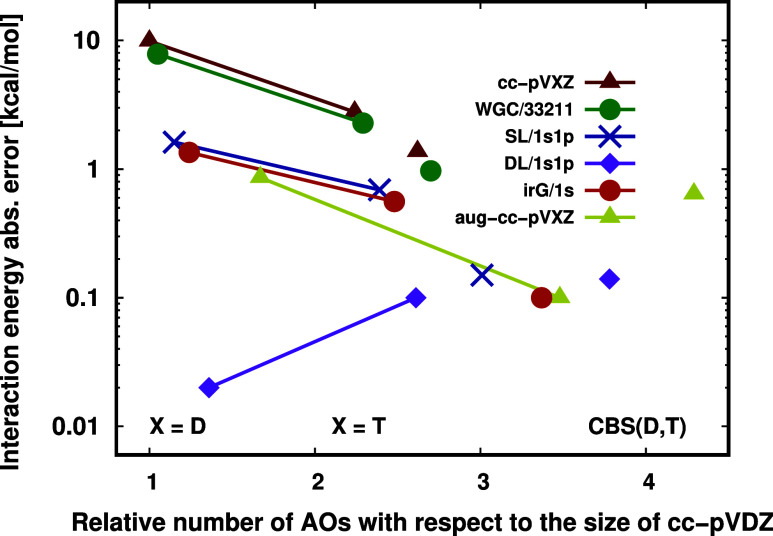
Absolute interaction energy errors of the LNO–CCSD(T)/(aug-)cc-pVXZ (X = D, T) for the CP corrected correlation energy contributions on a logarithmic scale. Test system: parallelly displaced coronene dimer. Reference: CP corrected LNO–CCSD(T)/aug-cc-pV(Q,5)Z.^54^

We generated the FO center lists of various FO methods (see, e.g., the irG and DL FO centers in Figure 9a,b) to investigate their effect on the basis set convergence of the coronene dimer interaction energy. To that end, LNO–CCSD(T)/cc-pVXZ (X = D, T) interaction energies with Tight LNO thresholds (Figure 10 and Table 3) were compared to our aug-cc-pV(Q,5)Z CBS reference computations from ref (54).

**Table 3:** LNO–CCSD(T)/(aug-)cc-pVXZ (X = D, T) Interaction Energy Errors of the Parallelly Displaced Coronene Dimer with Respect to the CP Corrected LNO–CCSD(T)/aug-cc-pV(Q,5)Z CBS Reference (54)^a^

AO basis	FO method (num. of centers)	(Rel.) num. of AOs + FOs	int. energy error [kcal/mol]	runtime (dimer) [h]
cc-pVDZ	–(0)	792 (1.0)	9.89	45.7
	WGC/33211 (1)	830 (1.1)	7.84	54.4
	SL/1s1p (36)	908 (1.2)	1.62	81.9
	irG/1s (191)	983 (1.2)	1.35	90.9
	DL/1s1p (72)	1080 (1.4)	0.02	126.4
	msG/1s (422)	1214 (1.5)	0.08	125.6
cc-pVTZ	– (0)	1776 (2.2)	2.78	145.4
	WGC/33211 (1)	1814 (2.3)	2.28	165.0
	SL/1s1p (36)	1892 (2.4)	0.69	188.8
	irG/1s (191)	1967 (2.5)	0.56	172.0
	DL/1s1p (72)	2064 (2.6)	0.10	209.4
	msG/1s (422)	2198 (2.8)	0.05	209.0
cc-pV(D,T)Z	–(0)		–1.37	
	WGC/33211 (1)		–0.97	
	SL/1s1p (36)		0.15	
	irG/1s (191)		0.10	
	DL/1s1p (72)		0.14	
	msG/1s (422)		0.03	
aug-cc-pVDZ	–(0)	1320 (1.7)	0.86	82.6
aug-cc-pVTZ	–(0)	2760 (3.5)	–0.10	206.1
aug-cc-pV(D,T)Z	–(0)		–0.64	

a Local correlation threshold: Tight. The cc-pVDZ (cc-pVTZ) calculations were executed on 24 (32) CPU cores, and the aug-cc-pVDZ (aug-cc-pVTZ) calculation was executed on 32 (40) CPU cores.

Inspecting Figure 10, we find the pure AO basis and WGC results similar (cf. red and green curves). The reason is that compared to the extended size of the coronene dimer, the effect of a single FO center even with 38 extra functions is not notable. Hence, the time of the computation, as well as the number of the basis functions, do not change considerably as well when adding the WGC FOs to either cc-pVDZ or cc-pVTZ (see the corresponding columns of Table 3 for the numerical results). With the irG method (orange in Figure 10), the errors of the interaction energy improve by almost 1 order of magnitude with respect to the pure cc-pVDZ AO basis results. It requires only 1.2 times increase in the total AO and FO basis set size (relative to cc-pVDZ) and ca. twice as long computation time. This finding also holds for the SL/1s1p FO method (blue in Figure 10) with not notably fewer basis functions with respect toirG/1s. Both the SL/1s1p and irG/1s numerical performances are similar to the pure aug-cc-pVDZ basis (yellow in Figure 10), but the number of basis functions used for the aug-cc-pVDZ is ca. 1.5 times larger than that of the SL/1s1p or irG/1s FO methods.

The performance of the cc-pVDZ + DL/1s1p FO method (purple in Figure 10) is better than that of irG/1s and SL/1s1p, as even 0.1 kcal/mol accuracy is surpassed, which is a 2 orders of magnitude improvement in the interaction energy relative to the pure cc-pVDZ error. Considering the 0.1 kcal/mol error of cc-pVTZ + DL/1s1p and cc-pV(D,T)Z CBS results, the excellent cc-pVDZ + DL/1s1p performance should be interpreted as partly fortunations. While cc-pVTZ + DL/1s1p considerably outperforms cc-pVTZ + irG/1s and cc-pVTZ + SL/1s1p, their CBS extrapolated results are all great, showing ca. 0.1 kcal/mol remaining BSIE. Moreover, cc-pVTZ + DL/1s1p is highly competitive with the pure aug-cc-pVTZ AO basis set, as both exhibit only 0.1 kcal/mol absolute BSIE, but the aug-cc-pVTZ basis set contains almost 1.4 times more basis functions. Due to the planar and parallel structure of the coronene dimer (Figure 9), all dimer atoms were added to the interacting surface atom list. Thus, with the DL method, 72 FO centers were positioned in the interacting region and thus 1 set of s and p FOs are assigned to each atoms. We note that further increasing the FO basis to 1s1p1d resulted in a near-linear dependency of the basis set, which originates from the planar structure of the monomers and the highly ordered alignment of the corresponding AOs and FOs. Therefore, we did not perform further investigations with DL/1s1p1d on the coronene dimer, while it could still be a useful approach for large but not as symmetric molecular dimers.

Turning to the analysis of the msG method (see Table 3), its numerical performance is similar to that of DL/1s1p in terms of both accuracy and computation time as it contains only 134 more FOs than DL/1s1p. Furthermore, we can also observe that the improvement is larger going from the pure cc-pVXZ basis set to cc-pVXZ + irG/1s than from cc-pVXZ + irG/1s to cc-pVXZ + msG/1s. This finding also corroborates that the FO centers outside of the interacting region bring less significant improvement to the interaction energies, than the 45% of the msG FOs kept in the interacting region when constructing irG. All in all, the performance of DL/1s1p and msG/1s with both cc-pVDZ and cc-pVTZ (as well as of irG/1s with cc-pVTZ) are remarkable, being in the tenths of a kcal/mol BSIE range. Since we cannot generalize far from these promising results obtained for one challenging system, the broader investigation of large noncovalent dimers is needed and planned in the future.

We note that Tight LNO thresholds were selected for the coronene dimer investigations to suppress the local approximation error to or below the range of BSIE. Therefore, due to the increased number of operations induced by the Tight LNO threshold, as well as the complicated long-range π–π interactions in the coronene dimer, the calculations are more time-consuming than the average for 50–100 atom molecules.^48^ Compared to that, LNO–CCSD(T)/cc-pVDZ (cc-pVTZ) calculations with normal LNO settings, without FOs, took 13.3 (37.2) h, while DL/1s1p with the same theoretical level and LNO threshold took 24.0 (57.6) h on the dimer, both with only 8 CPU cores.

## 5 Conclusions and Outlook

In this study, we systematically compared previous non-atom-centered or floating orbital (FO) basis approaches^65,79^ with here proposed novel FO methods for medium-sized (H-bond, ionic H-bond, dispersion, and mixed) molecular complexes,^81,82^ as well as on a large-scale application (coronene dimer). The so far almost exclusively employed approach uses a single FO center in the space between the two interacting monomers combined with a relatively large basis (up to 38 FOs) placed on that center.^65^ While even a single FO center can decrease the double-ζ AO basis set errors by 50–60%, e.g., for dispersion dominated dimers of ca. 20–30 atoms, the analogous improvement for more extended molecules is much smaller (e.g., ca. 20% for the coronene dimer). Overcoming some of the limitations of using a single FO center, a recent alternative method places a grid of FO centers and a single s type function per grid point onto the surface of the monomers.^79^

Here, we first showed that there is no need for completely surrounding the monomers with FOs, and their use can be limited to the space between the noncovalently interacting monomers. This resulted in the use of up to 1.5 times less AO and FO basis functions altogether compared to the FO grid method completely surrounding the monomers with a negligible loss of accuracy. Furthermore, we introduced a novel FO method which strategically adds one-one layer of FO centers onto the surface of each monomer facing the other monomer, thereby adding ca. one FO center to each atom that plays a key role in the interaction. With this more compact FO center list, we could employ more than a single s function on this double layer of FO centers. Moreover, this new double layer approach is considerably more general, regarding some limitations of the previous methods. For example, it performs much better for large molecules than the single FO center and does not have the limitation of the previous FO center grid method being optimized only for H, C, N, and O atoms.^79^

Our statistical analysis showed that the most beneficial choice is to use a set of s and p functions (1s1p) or an additional set of d functions (1s1p1d) per FO center in the new double layer FO method. For example, for smaller, H-bonded systems, cc-pVDZ with the 1s1p1d FO basis could outperform (reach) the accuracy of the pure cc-pVTZ (aug-cc-pVDZ) AO basis with 1.6 (1.1) times fewer (AO and FO) basis functions. For the more dispersion dominated complexes with larger interacting surfaces, even the accuracy of the pure aug-cc-pVTZ is approached (with 2.1 times fewer orbitals), while with cc-pVDZ even the smaller 1s1p FO basis had pleasing performance.

Therefore, the proposed FO method can successfully replace diffuse AOs or decrease the cardinal number of the AO basis, both of which are particularly helpful for large molecules to ease the computational cost and frequent near-linear-dependency issues. Additionally, the number of FOs added to the interacting surface scales much more favorably with the system size than adding, e.g., diffuse AO onto all atoms. As presented for the complicated interactions in the coronene dimer, the combination of FO methods with efficient asymptotically linear-scaling local correlation methods, such as our local natural orbital LNO–CCSD(T),^43,48,52^ can make large-scale interaction energy computations accurate and routinely accessible. In particular for the coronene dimer, adding an FO extension of ca. 40% of the size of cc-pVDZ with our novel FO method could decrease the 10 (3) kcal/mol basis set error of the cc-pVDZ (cc-pVTZ) interaction energies to ca. 0.1 kcal/mol compared to the expensive LNO–CCSD(T)/aug-cc-pV(Q,5)Z CBS extrapolated reference.^54^

Considering that our LNO–CCSD(T) method was applicable to compute protein–ligand interaction energies using more than a 1000 atoms and a quadruple-ζ AO basis set,^43,48^ the presented FO method development can notably extend the scope of accurate and routinely accessible noncovalent interaction computations. For example, LNO–CCSD(T) with triple-ζ AO and the proposed FO basis should be routinely applicable for a few hundred atoms with a single CPU and few 10 GBs of memory, covering a wide range of supramolecular, catalyst-substrate, drug–protein active site, solute–solvent, surface adsorption, etc. interactions, which will be further demonstrated also in our forthcoming studies.

## Supporting Information Available

Complete list of the calculated HF interaction energies and the DF-MP2, and if available, DF-CCSD(T), LMP2, LNO–CCSD, and LNO–CCSD(T) correlation energy contributions of the interaction energies (XLS)
Mrcc input file (MINP) samples for the DF-CCSD(T) calculation of the S66 uracil dimer, serial number 26; Mrcc basis set file (GENBAS) collecting the utilized floating orbital basis sets, contraction coefficients, and exponents (ZIP)
Complete list of the names and the Cartesian coordinates employed for the S66 and IHB100/21 complexes, as well as for the parallelly displaced coronene dimer augmented with floating orbital positions (ZIP)
Further numerical data visualization regarding the accuracy assessment and comparison of the here introduced floating orbital methods (PDF)
