# Supplementary material for: Advancing Non-Atom-Centered Basis Methods for More Accurate Interaction Energies: Benchmarks and Large-Scale Applications
Source: J Phys Chem A. 2024 Nov 18;128(47):10282–98. doi: 10.1021/acs.jpca.4c04689 (PMC11613648; doi:10.1021/acs.jpca.4c04689)
Supplement: Supplementary file 4 — jp4c04689_si_004.pdf [file jp4c04689_si_004.pdf]

Supporting Information to Advancing Non-Atom-Centered Basis Methods for More Accurate Interaction Energies: Benchmarks and Large-Scale Applications

Balázs D. Lőrincz[†,‡,¶] and Péter R. Nagy[*,†,‡,¶]

†*Department of Physical Chemistry and Materials Science, Faculty of Chemical Technology and Biotechnology, Budapest University of Technology and Economics, Műegyetem rkp. 3., H-1111 Budapest, Hungary*

‡*HUN-REN–BME Quantum Chemistry Research Group, Műegyetem rkp. 3., H-1111 Budapest, Hungary*

¶*MTA–BME Lendület Quantum Chemistry Research Group, Műegyetem rkp. 3., H-1111 Budapest, Hungary*

E-mail: nagy.peter@vbk.bme.hu

S1 Interaction energy errors for various FO method and AO basis settings

Table S1: DF-MP2 and DF-CCSD(T) interaction energy basis set errors in kcal/mol (MP2 and CCSD(T) columns, respectively) for uracil-uracil base pair (hydrogen bonded) and the $\pi - \pi$ stacking (dispersion) uracil dimer from the S66 test set with 6-31+G(2d) AO basis set. CBS reference: CP corrected MP2-F12/haug-cc-pV(T,Q)Z and DF-CCSD(T)/haug-cc-pV(T,Q).[1]

Uracil dimer	FO method (num. of centers)	(Rel.) num. of AOs + FOs	MP2	CCSD(T)
hydrogen bonded	– (0)	384 (1.5)	1.97	2.51
	WGC/33211 (1)	422 (1.6)	1.02	1.24
	SL/1s1p (4)	400 (1.5)	1.71	2.16
	SL/1s1p1d (4)	420 (1.6)	1.24	1.51
	DL/1s1p (8)	416 (1.6)	1.69	2.13
	DL/1s1p1d (8)	456 (1.7)	1.12	1.35
	irG/1s (37)	421 (1.6)	1.04	1.24
	msG/1s (159)	543 (2.1)	1.00	1.20
dispersion	– (0)	384 (1.5)	2.12	2.16
	WGC/33211 (1)	422 (1.6)	1.13	1.09
	SL/1s1p (12)	432 (1.6)	1.16	1.08
	SL/1s1p1d (12)	492 (1.9)	0.69	0.52
	DL/1s1p (24)	480 (1.8)	1.04	0.93
	DL/1s1p1d (24)	600 (2.3)	0.46	0.27
	irG/1s (78)	462 (1.8)	0.94	0.79
	msG/1s (172)	556 (2.1)	0.85	0.69

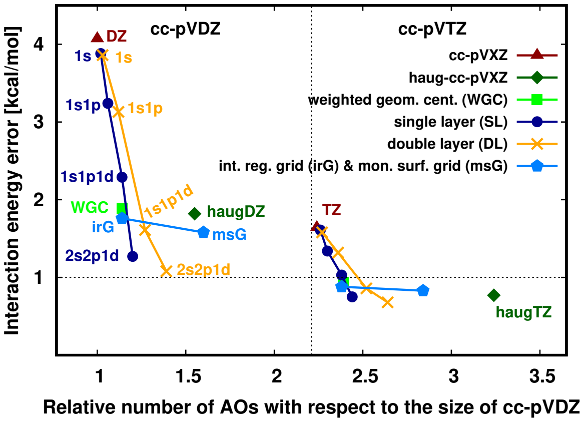

(a)

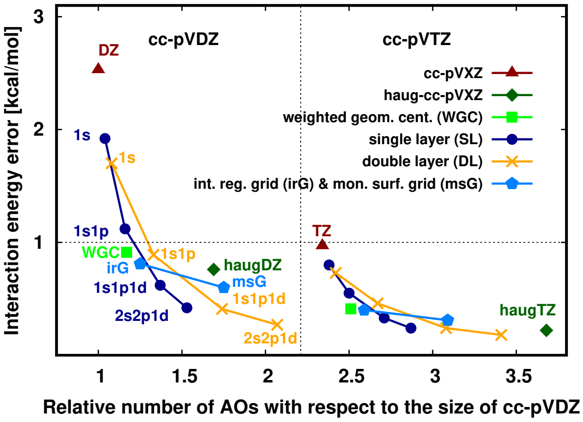

(b)

Figure S1: DF-CCSD(T)/cc-pVXZ (+FO) (X = D, T) correlation energy contribution error of CP corrected interaction energies as a function of the combined AO and FO basis set size for the hydrogen-bonded uracil dimer (a), as well as for the Benzene-Peptide dimer (b) of the S66 test set. Reference: CP corrected DF-CCSD(T)/haug-cc-pV(T,Q)Z.[1]

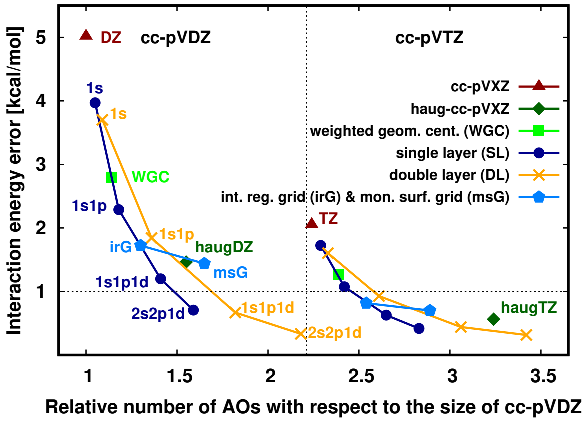

(a)

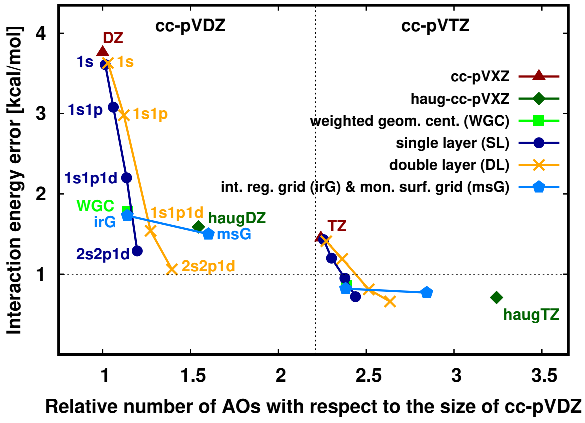

(b)

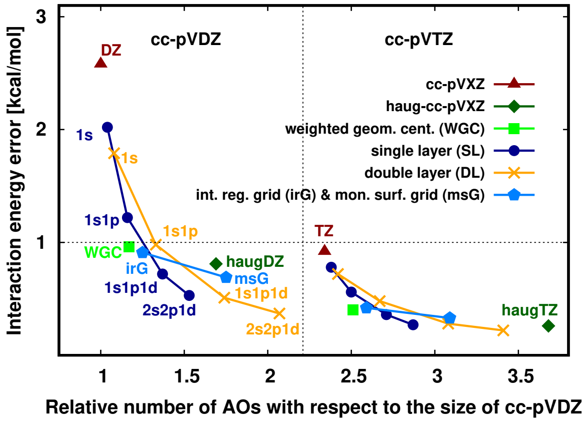

(c)

Figure S2: DF-MP2/cc-pVXZ (+FO) (X = D, T) correlation energy contribution error of CP corrected interaction energies as a function of the combined AO and FO basis set size for the dispersive uracil dimer (a), for the hydrogen-bonded uracil dimer (b), as well as for the Benzene-Peptide dimer (c) of the S66 test set. Reference: CP corrected MP2-F12/haug-cc-pV(T,Q)Z.[1]

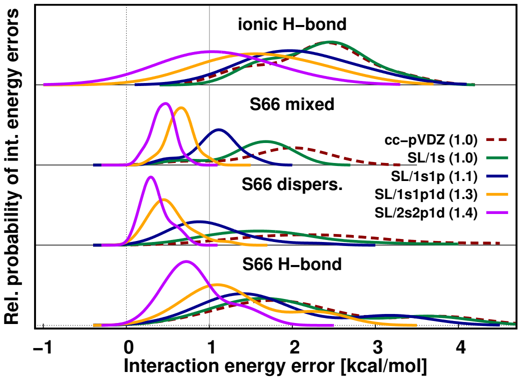

(a)

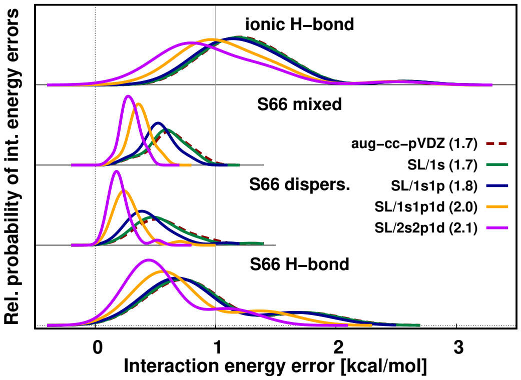

(b)

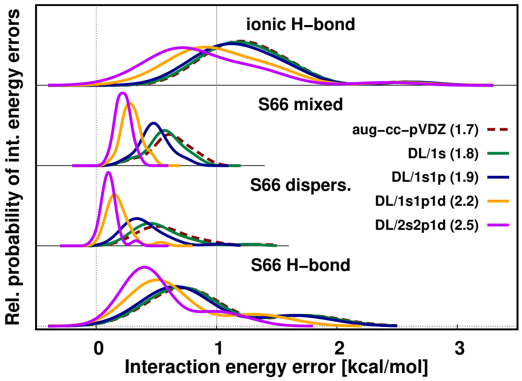

(c)

Figure S3: Relative probability of interaction energy errors with the SL and the DL FO method and 1s, 1s1p, 1s1p1d, and 2s2p1d FO bases. Level of theory: DF-MP2/(aug-)cc-pVDZ (SL + cc-pVDZ, a ; SL + aug-cc-pVDZ, b ; DL + aug-cc-pVDZ, c). The total number of basis functions relative to the size of cc-pVDZ is collected in parentheses besides the basis set labels.

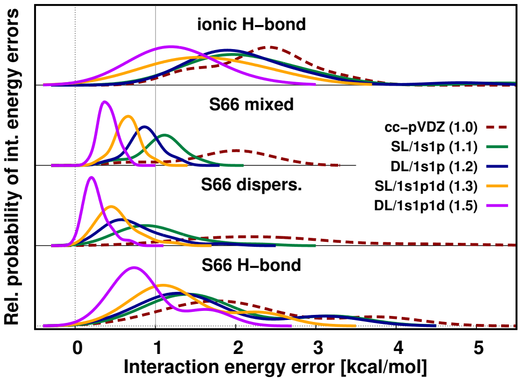

(a)

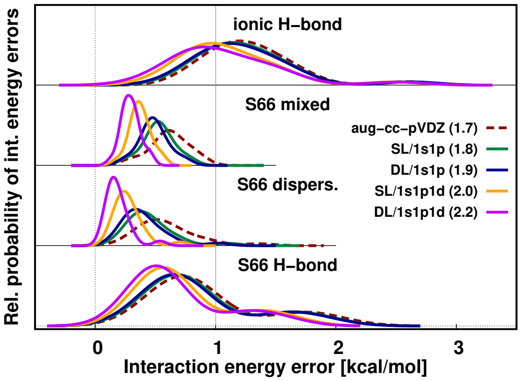

(b)

Figure S4: Relative probability of interaction energy errors with the SL and the DL FO method and 1s1p and 1s1p1d FO bases. Level of theory: DF-MP2/(aug-)cc-pVDZ (cc-pVDZ, a ; aug-cc-pVDZ, b). The total number of basis functions relative to the size of cc-pVDZ is collected in parentheses besides the basis set labels.

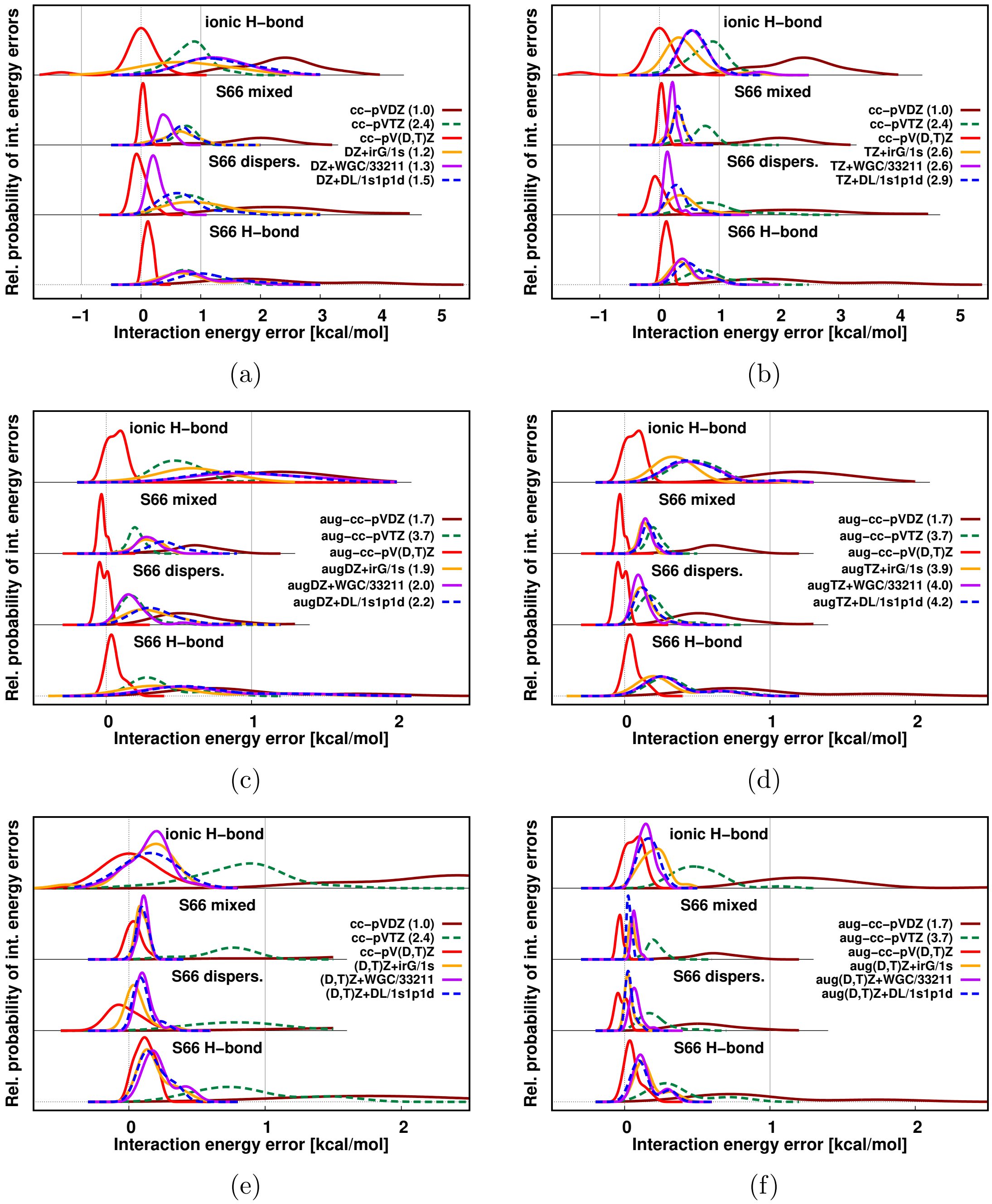

Figure S5: Relative probability of interaction energy errors at DF-MP2/(aug-)cc-pVXZ, X = D, T level of theory using pure AO basis sets and various FO methods added to cc-pVDZ (DZ, a), cc-pVTZ (TZ, b), aug-cc-pVDZ (augDZ, c), aug-cc-pVTZ (augTZ, d), cc-pV(D,T)Z [(D,T)Z, e], and aug-cc-pV(D,T)Z [aug(D,T)Z, f]. The total number of basis functions relative to the size of cc-pVDZ is collected in parentheses besides the basis set labels.

Table S2: Mean absolute basis set errors [kcal/mol] for the mixed subset of S66 (top) and the ionic H-bonds (bottom). Level of theory: DF-MP2/(aug-)cc-pVXZ; X = D, T.

	**DZ**	**augDZ**	**TZ**	**augTZ**	**(D,T)Z**	**aug(D,T)Z**
**mixed interactions of S66**						
AO basis	1.83	0.61	0.70	0.20	0.05	0.03
WGC/33211	0.64	0.29	0.29	0.14	0.09	0.05
SL/1s1p	1.07	0.53	0.47	0.19	0.12	0.01
SL/1s1p1d	0.65	0.37	0.30	0.17	0.10	0.05
DL/1s1p	0.87	0.49	0.40	0.19	0.13	0.02
DL/1s1p1d	0.41	0.29	0.22	0.15	0.11	0.07
irG/1s	0.68	0.41	0.31	0.17	0.10	0.03
msG/1s	0.53	0.35	0.26	0.16	0.10	0.04
**ionic H-bonds**						
AO basis	2.30	1.30	0.81	0.52	0.15	0.07
WGC/33211	0.95	0.65	0.43	0.37	0.19	0.20
SL/1s1p	2.25	1.26	0.81	0.51	0.13	0.09
SL/1s1p1d	1.84	1.12	0.68	0.50	0.15	0.14
DL/1s1p	2.17	1.24	0.84	0.51	0.09	0.09
DL/1s1p1d	1.41	1.08	0.63	0.49	0.18	0.15
irG/1s	1.42	1.02	0.58	0.47	0.21	0.16
msG/1s	1.09	0.96	0.51	0.46	0.19	0.17

References

(1) Nagy, P. R.; Gyevi-Nagy, L.; Lőrincz, B. D.; Kállay, M. Pursuing the basis set limit of CCSD(T) non-covalent interaction energies for medium-sized complexes: case study on the S66 compilation. *Mol. Phys.* **2023**, *121*, e2109526.
